# Supplementary figures and images for: Erythropoietin alleviates lung ischemia-reperfusion injury by activating the FGF23/FGFR4/ERK signaling pathway (part 1 of 2)
Source: PeerJ. 2024 Mar 27;12:e17123. doi: 10.7717/peerj.17123 (PMC10981413; doi:10.7717/peerj.17123)

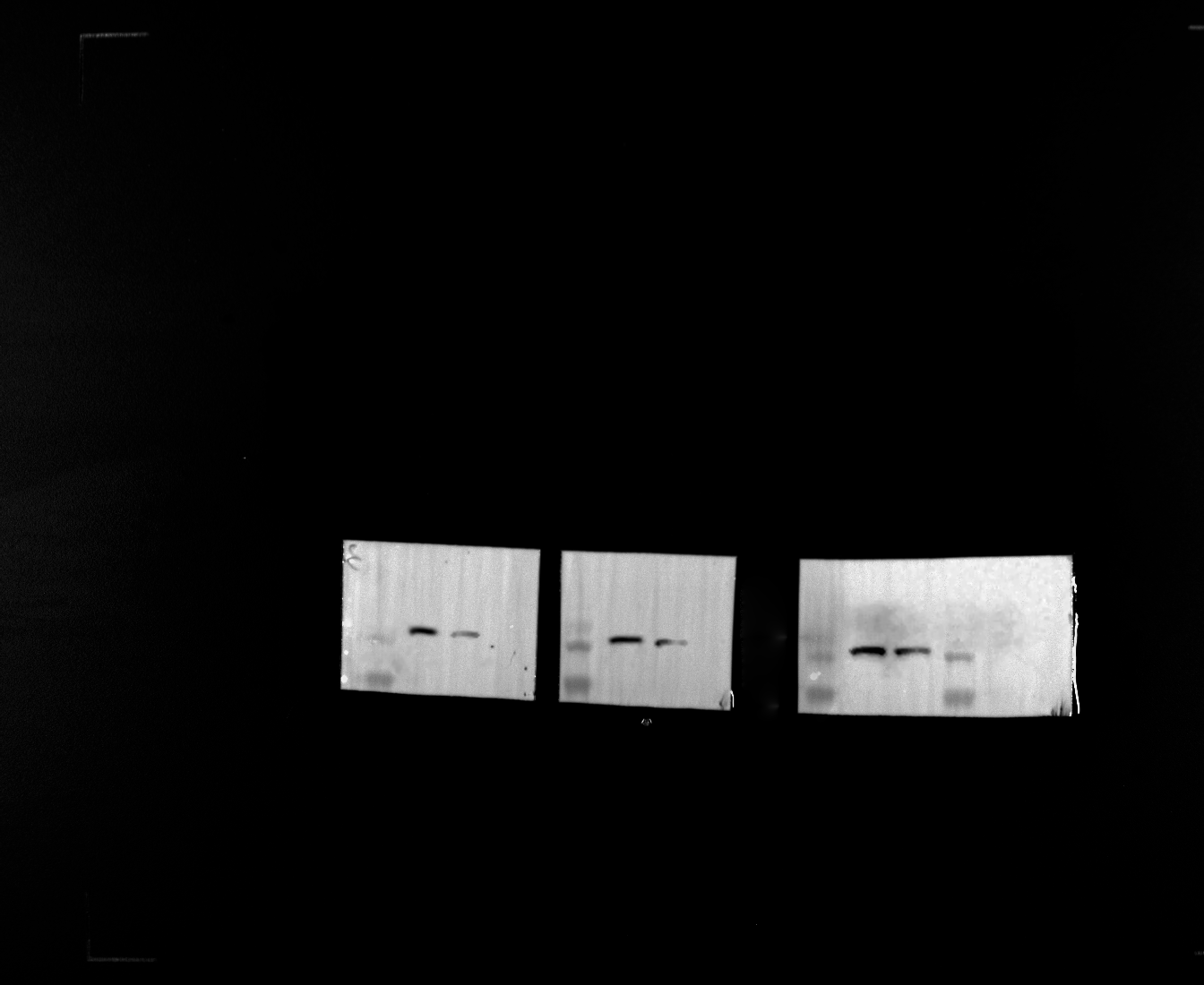

Supplement: Supplemental Information 1 [file peerj-12-17123-s001.zip › Fig1E blot/FGFR4/FGFR4-1、2、3-1.tif]

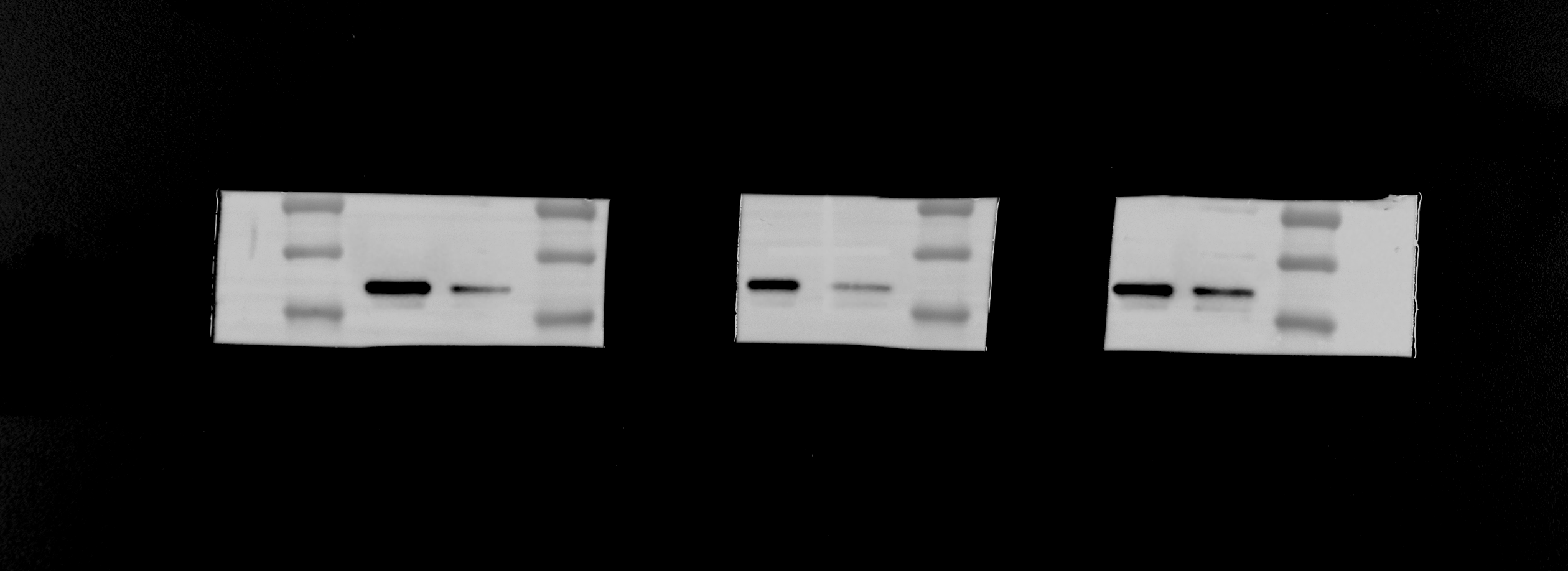

Supplement: Supplemental Information 1 [file peerj-12-17123-s001.zip › Fig1E blot/FGF23/FGF23-1、2、3.tif]

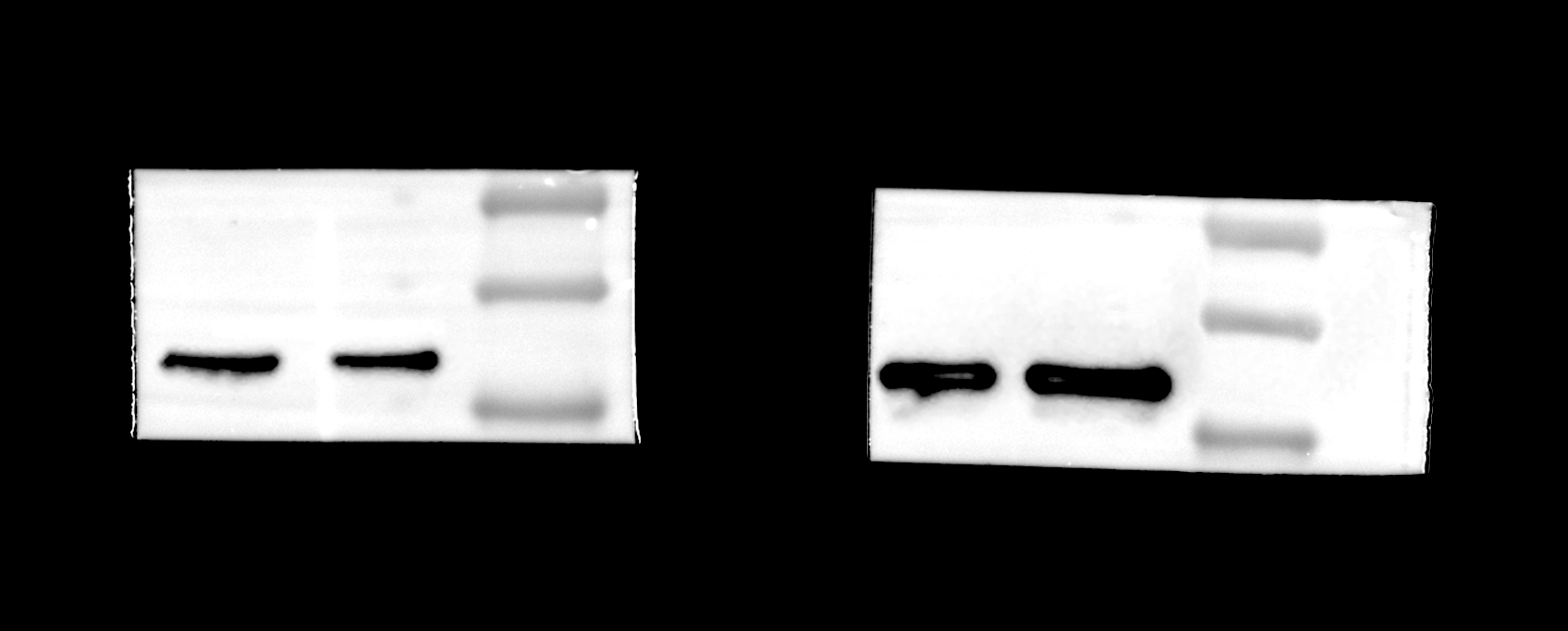

Supplement: Supplemental Information 1 [file peerj-12-17123-s001.zip › Fig1E blot/GAPDH/GAPDH-1、2.tif]

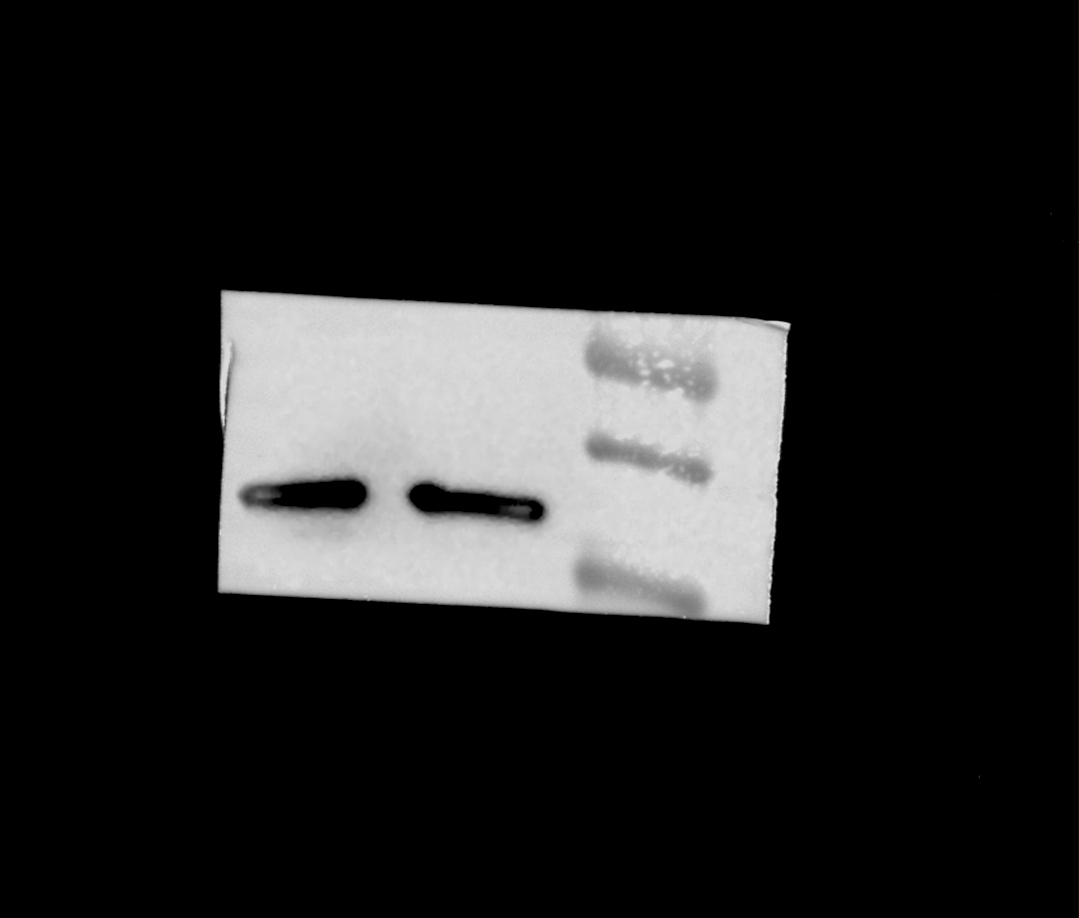

Supplement: Supplemental Information 1 [file peerj-12-17123-s001.zip › Fig1E blot/GAPDH/GAPDH-3.tif]

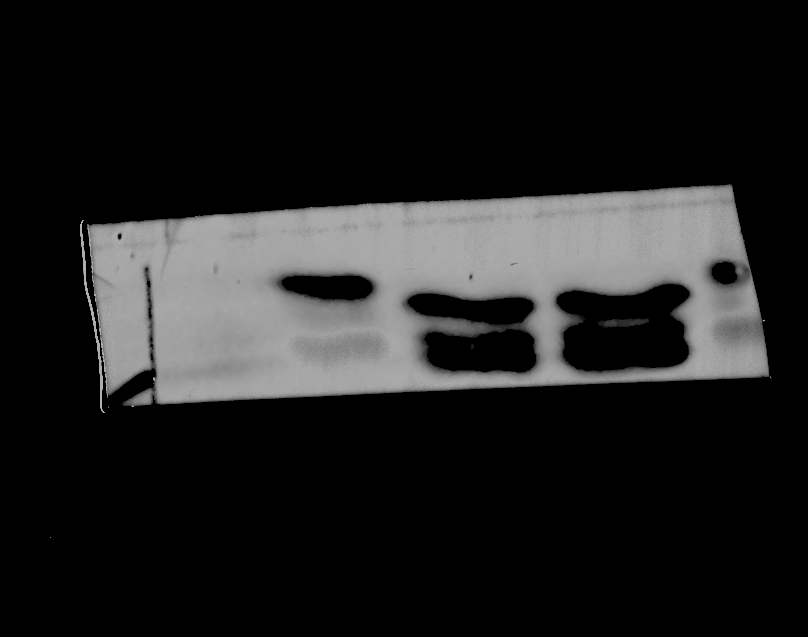

Supplement: Supplemental Information 1 [file peerj-12-17123-s001.zip › Fig1E blot/erk/erk-1.tif]

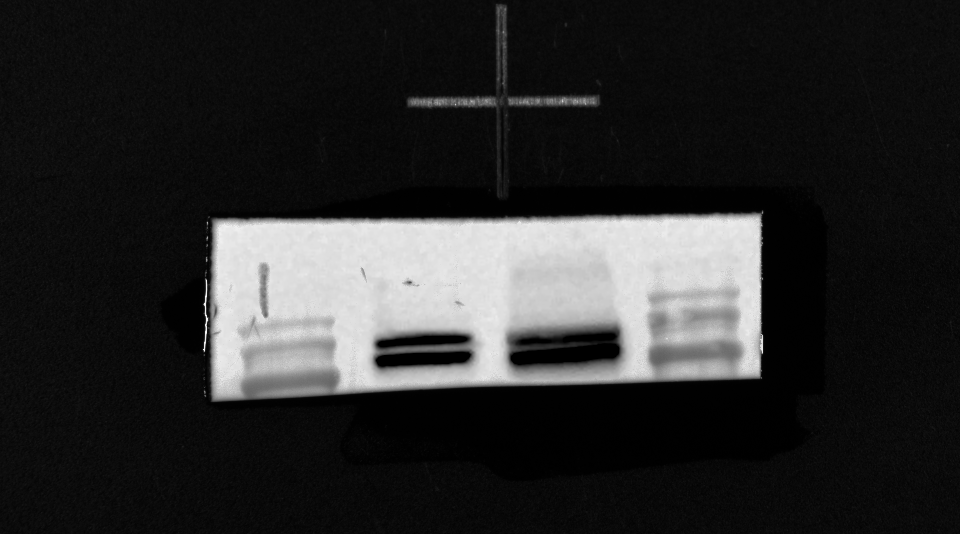

Supplement: Supplemental Information 1 [file peerj-12-17123-s001.zip › Fig1E blot/erk/erk-2.tif]

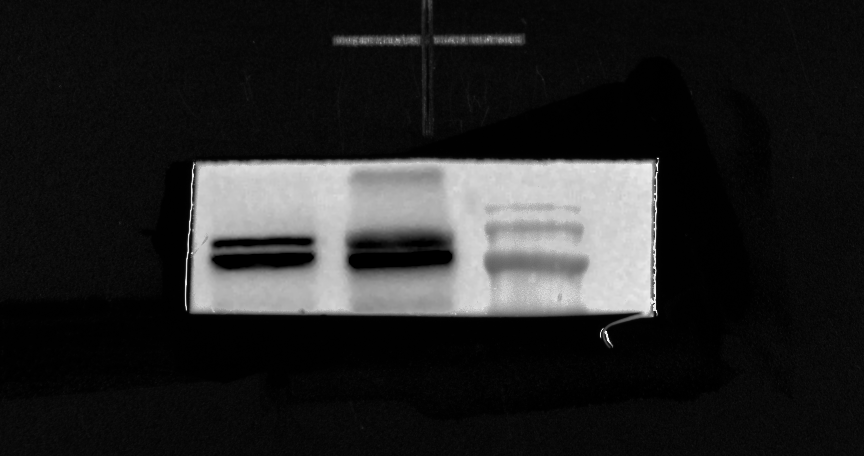

Supplement: Supplemental Information 1 [file peerj-12-17123-s001.zip › Fig1E blot/erk/erk-3.tif]

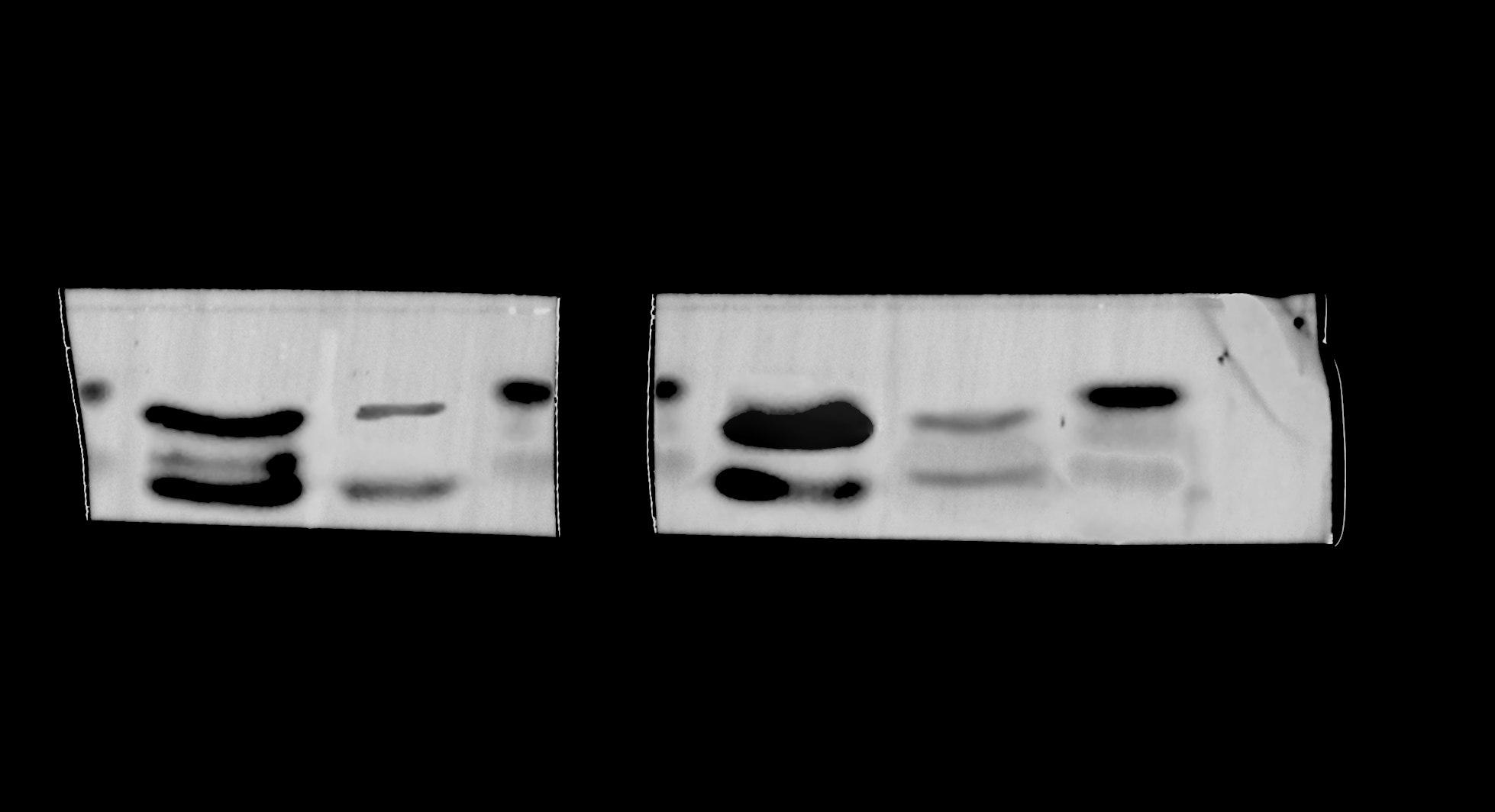

Supplement: Supplemental Information 1 [file peerj-12-17123-s001.zip › Fig1E blot/erk/p-erk-1、2.tif]

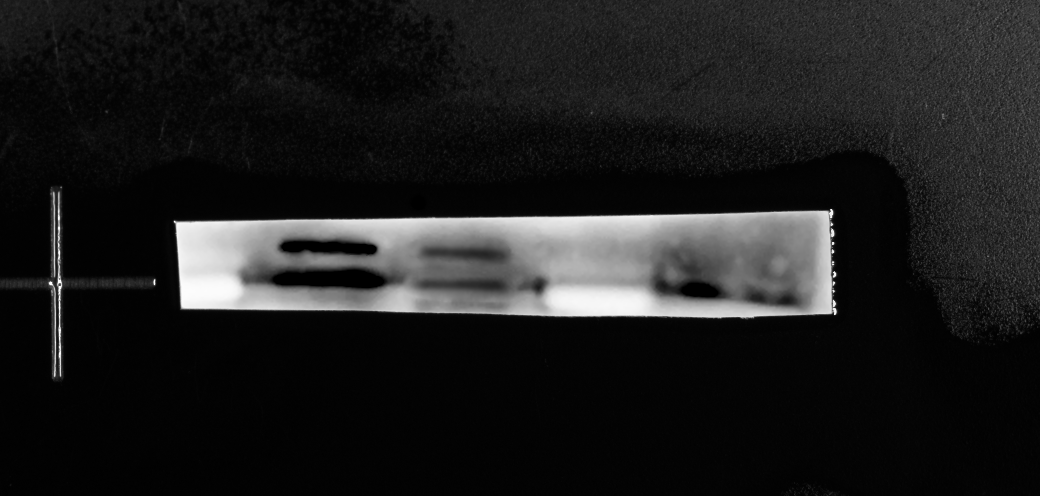

Supplement: Supplemental Information 1 [file peerj-12-17123-s001.zip › Fig1E blot/erk/p-erk-3.tif]

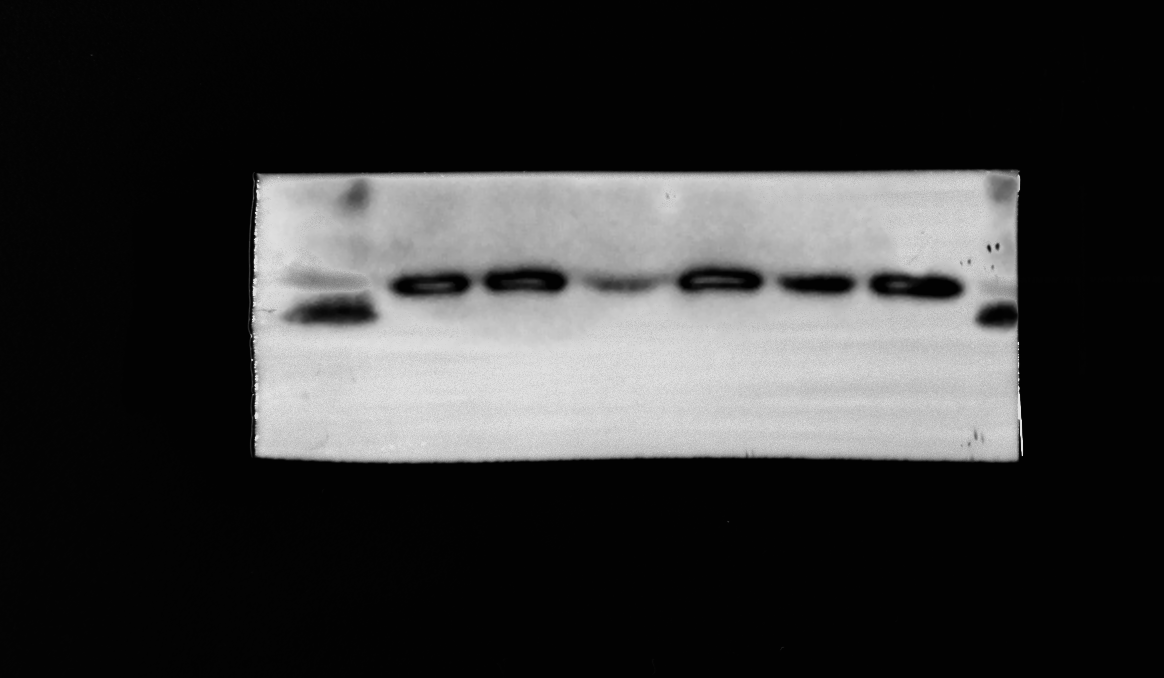

Supplement: Supplemental Information 1 [file peerj-12-17123-s001.zip › Fig2G blot/FGF23/FGF23-1.tif]

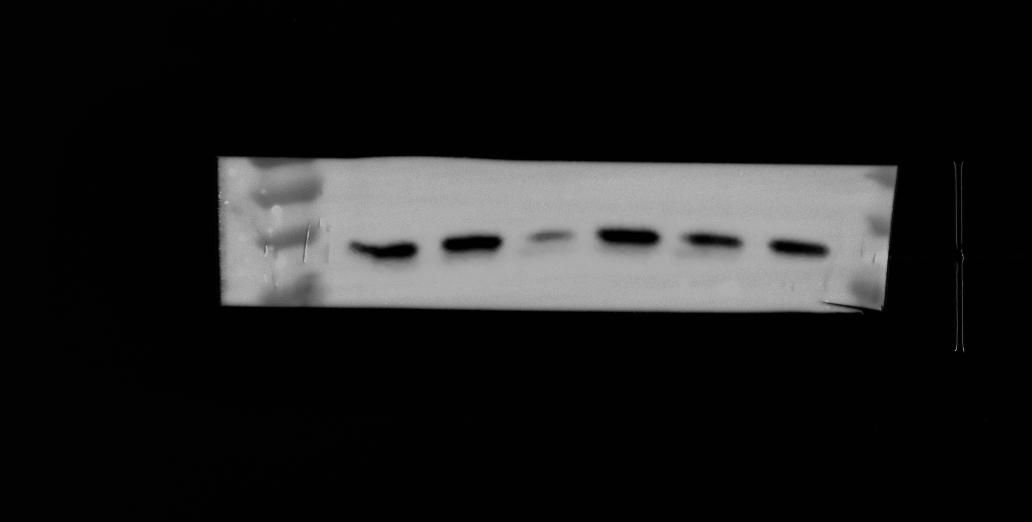

Supplement: Supplemental Information 1 [file peerj-12-17123-s001.zip › Fig2G blot/FGF23/FGF23-2.tif]

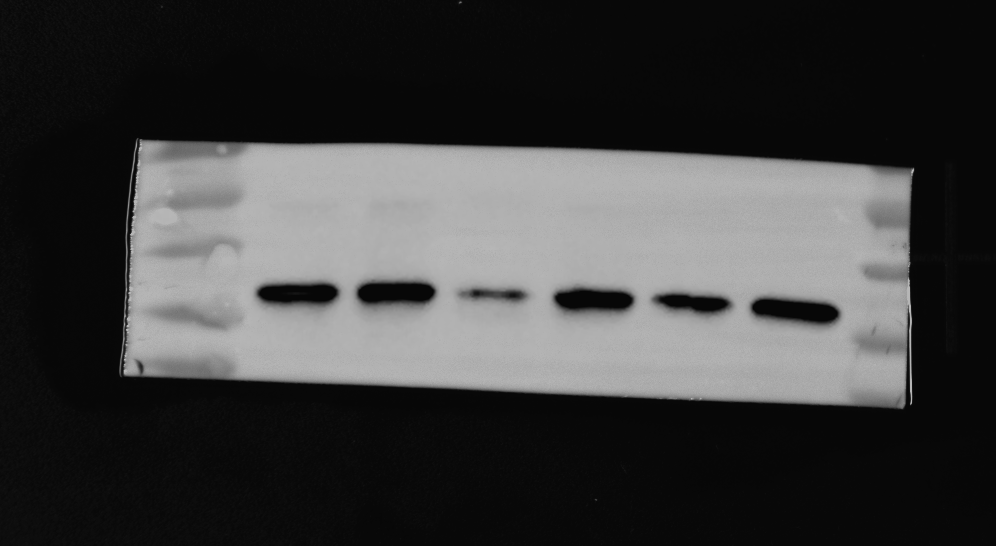

Supplement: Supplemental Information 1 [file peerj-12-17123-s001.zip › Fig2G blot/FGF23/FGF23-3.tif]

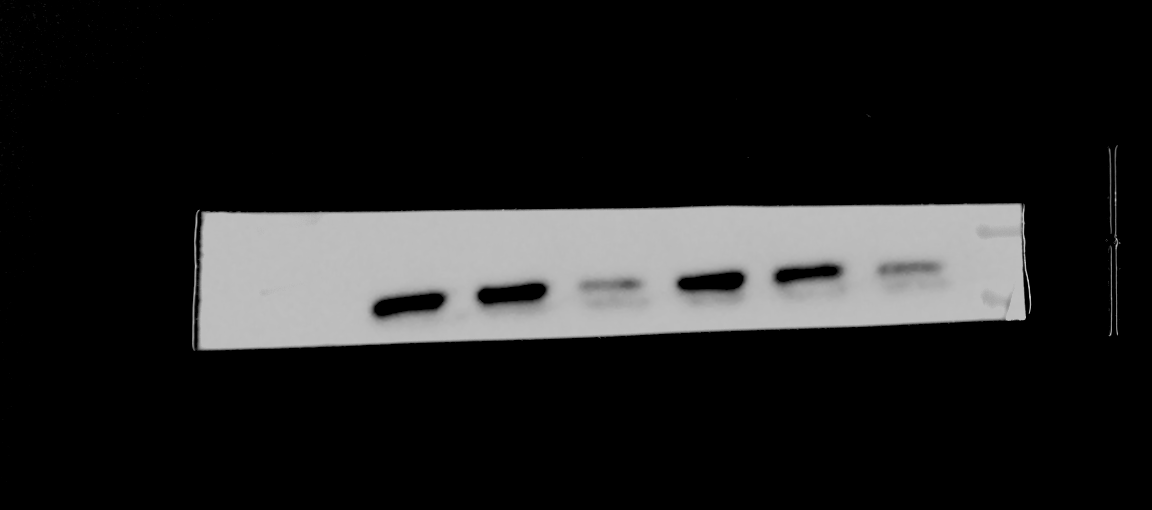

Supplement: Supplemental Information 1 [file peerj-12-17123-s001.zip › Fig2G blot/FGFR4/FGFR4-1.tif]

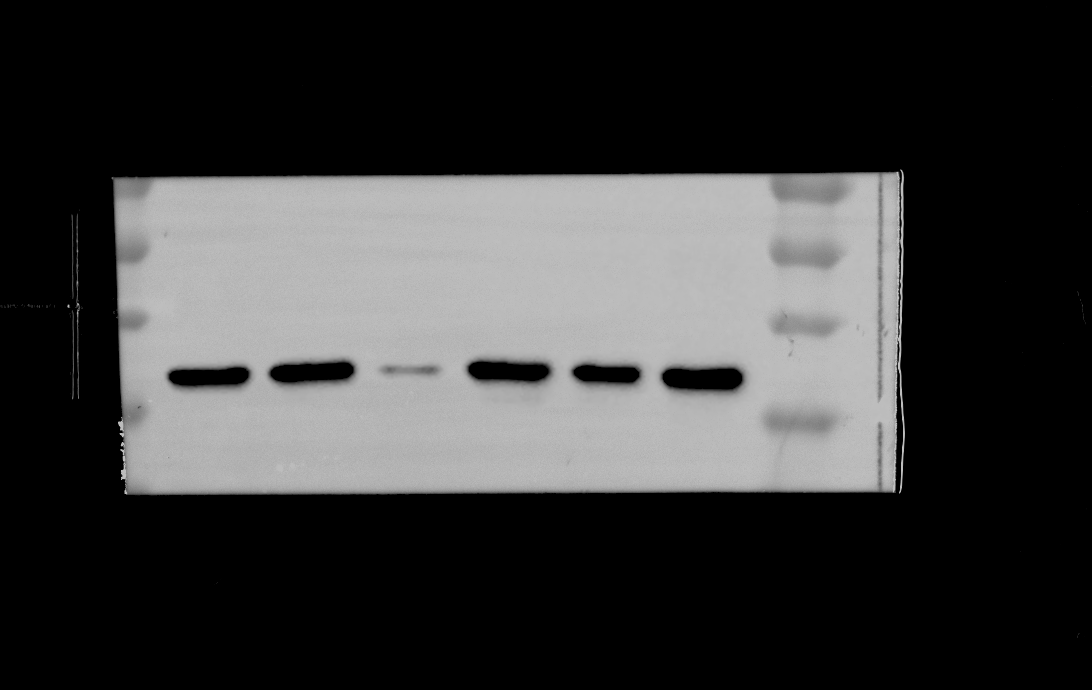

Supplement: Supplemental Information 1 [file peerj-12-17123-s001.zip › Fig2G blot/FGFR4/FGFR4-2.tif]

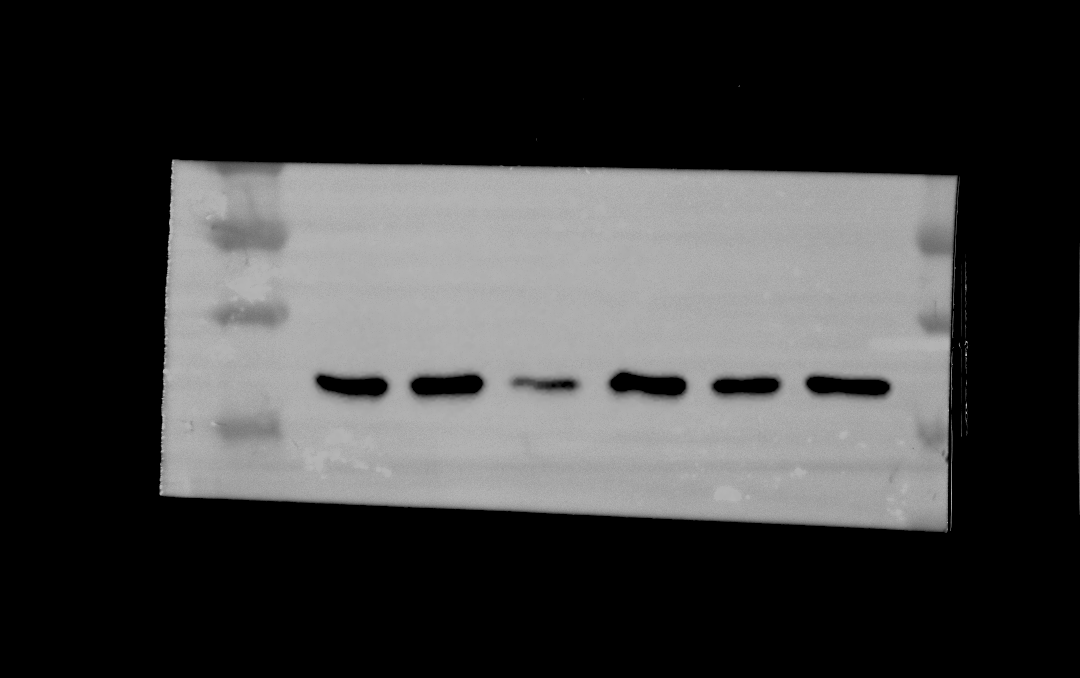

Supplement: Supplemental Information 1 [file peerj-12-17123-s001.zip › Fig2G blot/FGFR4/FGFR4-3.tif]

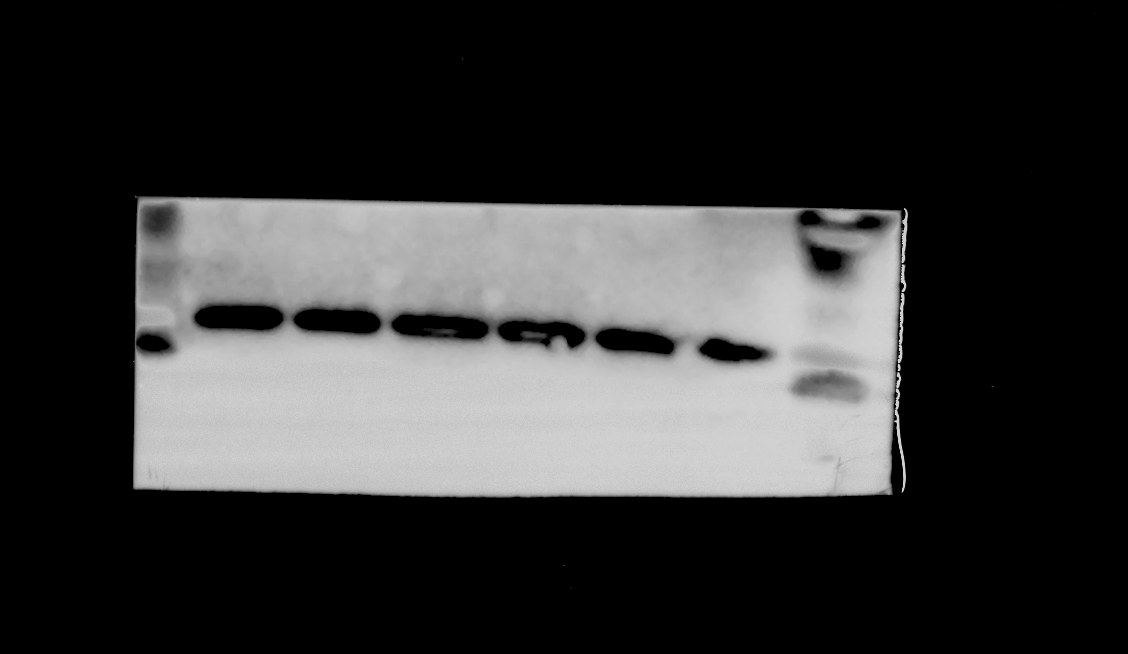

Supplement: Supplemental Information 1 [file peerj-12-17123-s001.zip › Fig2G blot/GAPDH/GAPDH-1.tif]

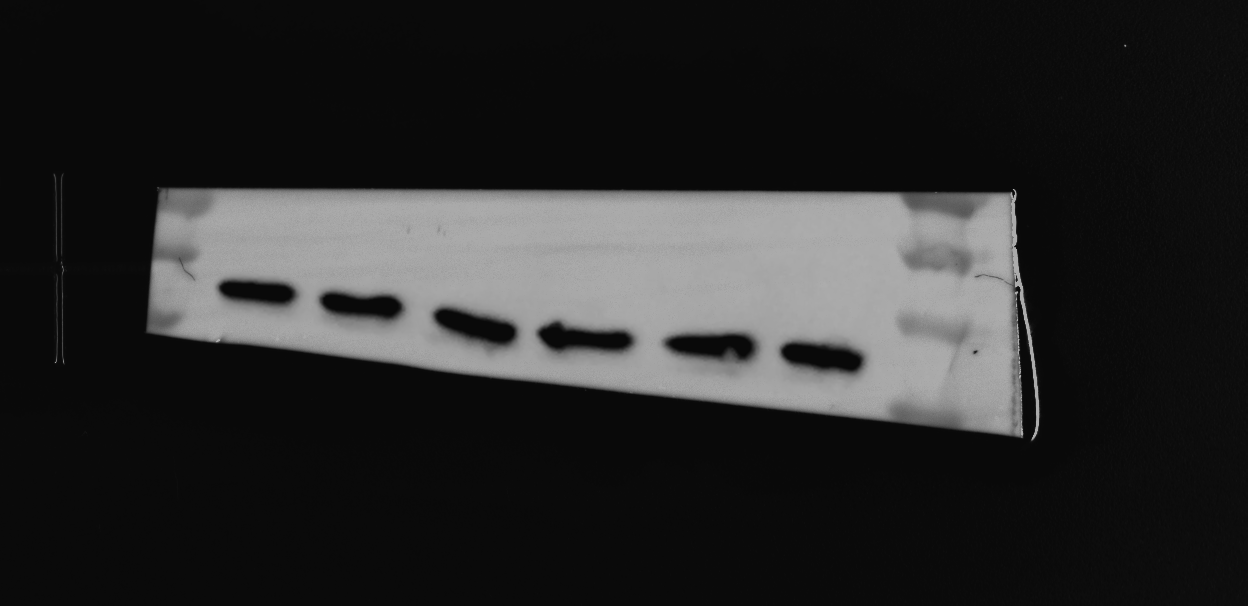

Supplement: Supplemental Information 1 [file peerj-12-17123-s001.zip › Fig2G blot/GAPDH/GAPDH-2.tif]

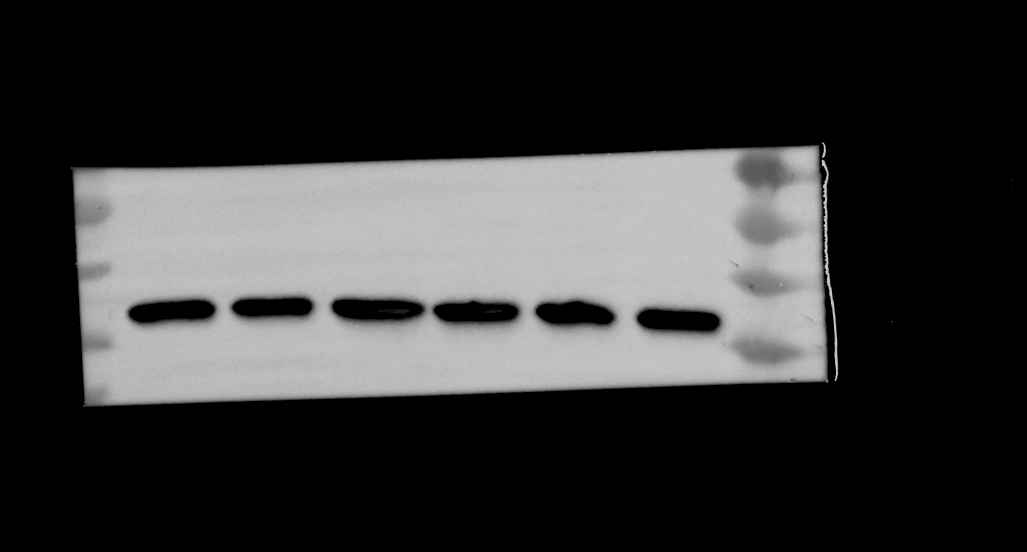

Supplement: Supplemental Information 1 [file peerj-12-17123-s001.zip › Fig2G blot/GAPDH/GAPDH-3.tif]

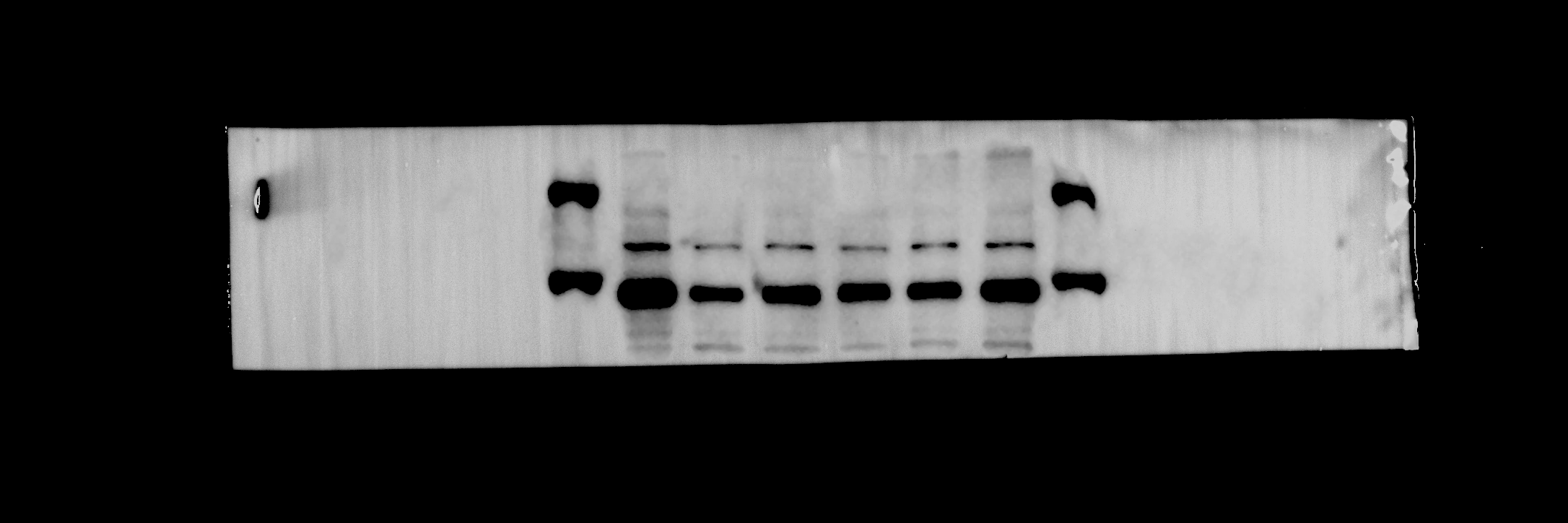

Supplement: Supplemental Information 1 [file peerj-12-17123-s001.zip › Fig2G blot/erk/erk-1.tif]

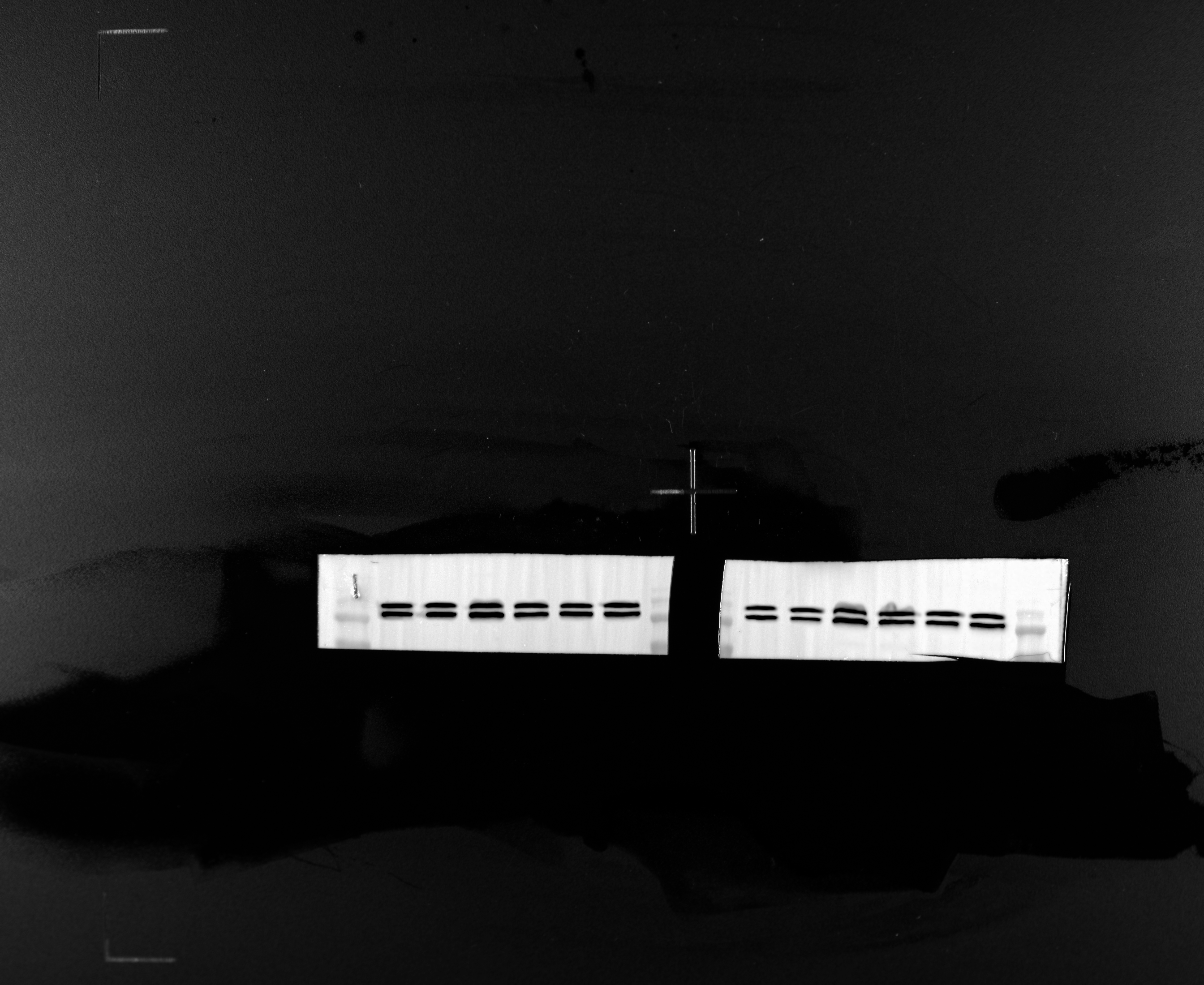

Supplement: Supplemental Information 1 [file peerj-12-17123-s001.zip › Fig2G blot/erk/erk-2、3.tif]

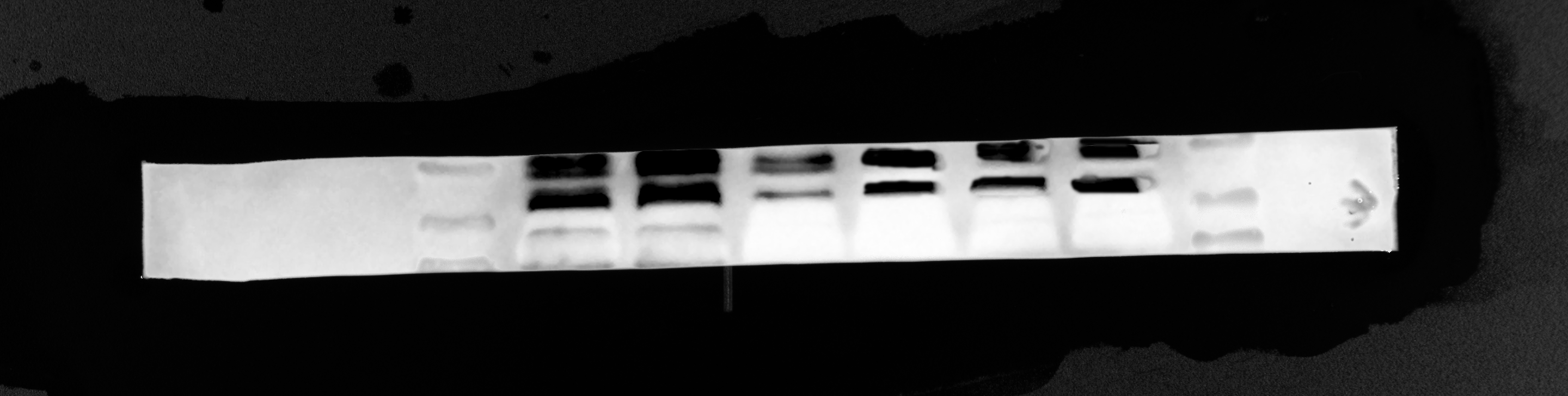

Supplement: Supplemental Information 1 [file peerj-12-17123-s001.zip › Fig2G blot/erk/p-erk-1.tif]

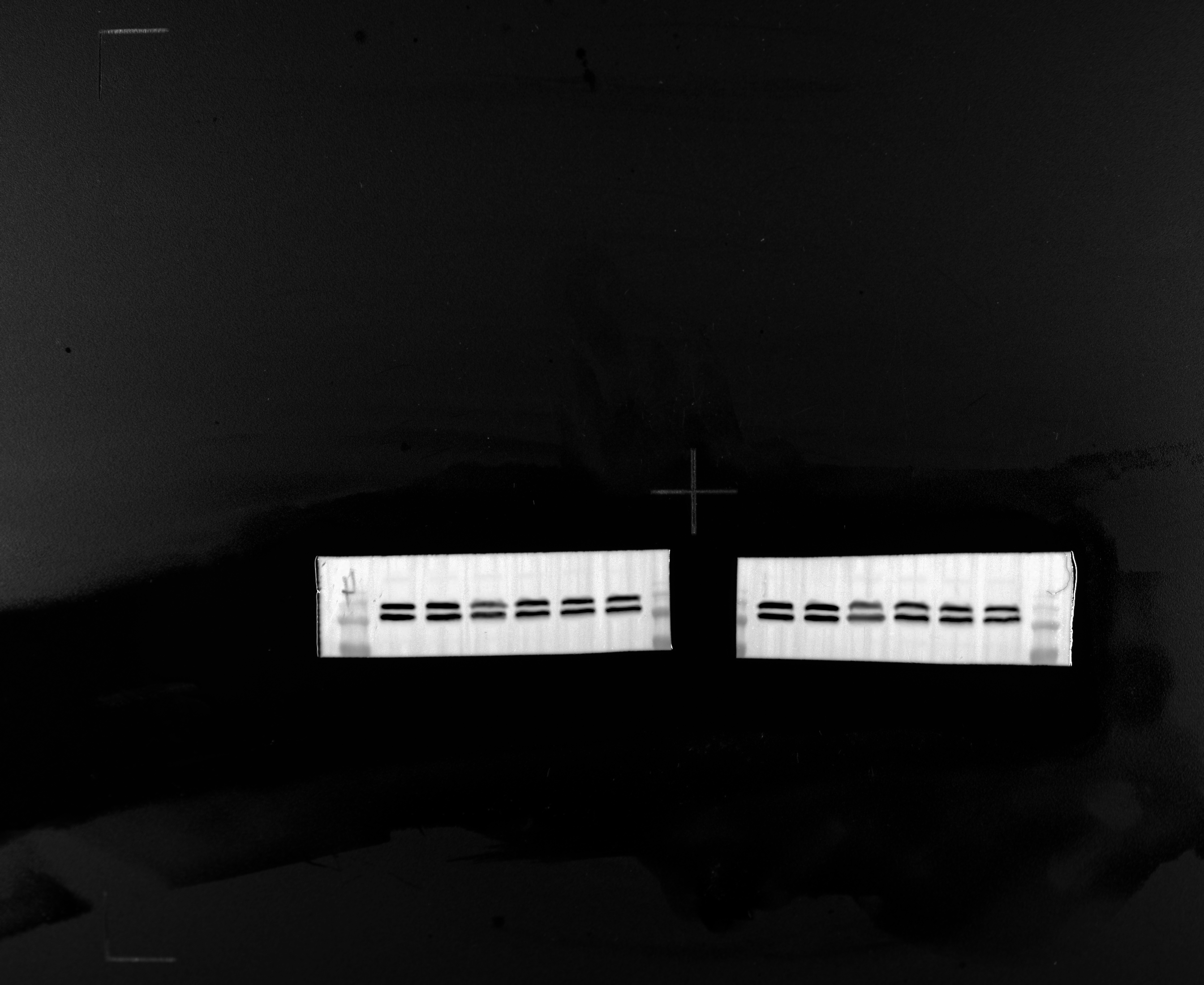

Supplement: Supplemental Information 1 [file peerj-12-17123-s001.zip › Fig2G blot/erk/p-erk-2、3.tif]

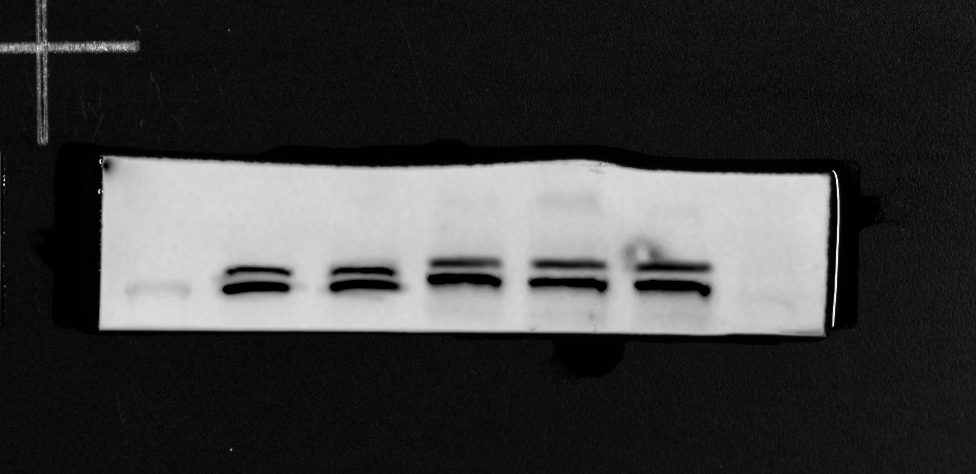

Supplement: Supplemental Information 1 [file peerj-12-17123-s001.zip › Fig3E blot/ERK/erk-1.tif]

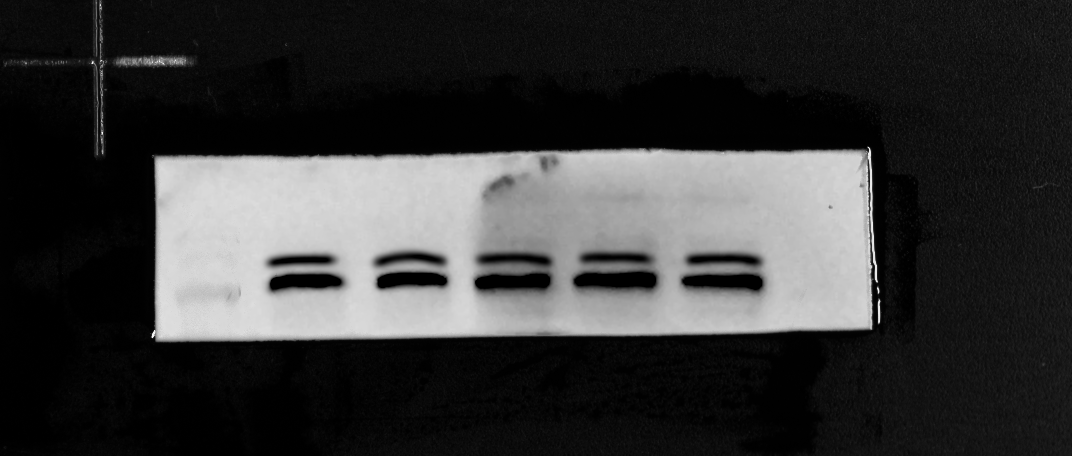

Supplement: Supplemental Information 1 [file peerj-12-17123-s001.zip › Fig3E blot/ERK/erk-2.tif]

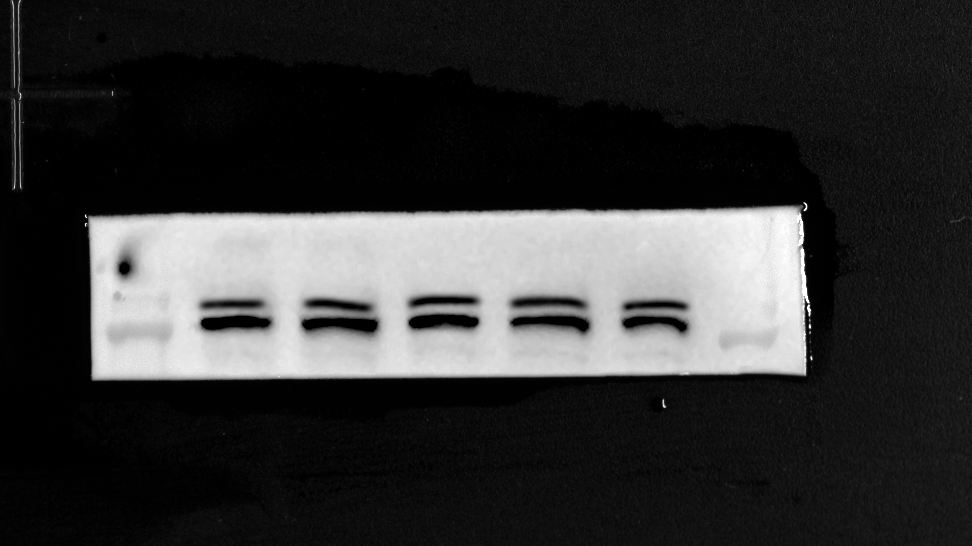

Supplement: Supplemental Information 1 [file peerj-12-17123-s001.zip › Fig3E blot/ERK/erk-3.tif]

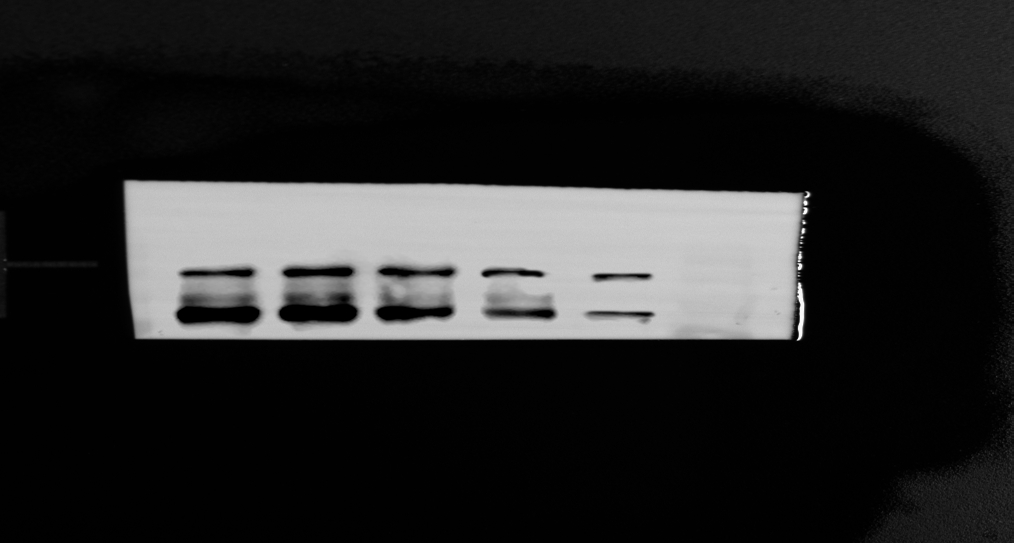

Supplement: Supplemental Information 1 [file peerj-12-17123-s001.zip › Fig3E blot/ERK/p-erk-1.tif]

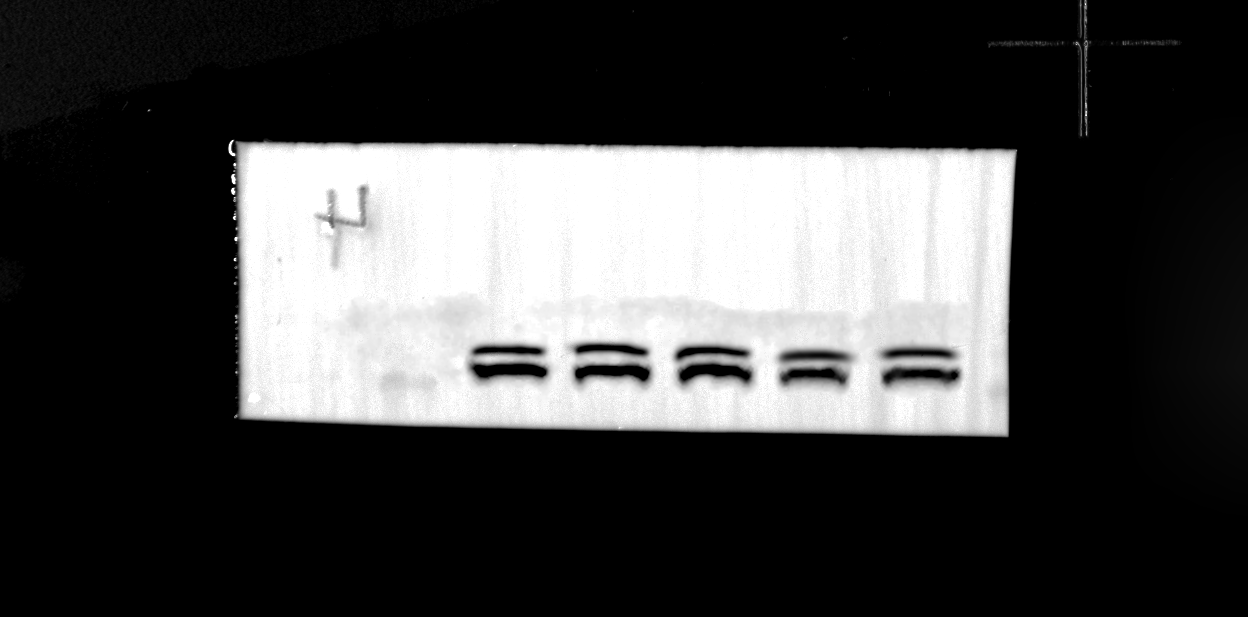

Supplement: Supplemental Information 1 [file peerj-12-17123-s001.zip › Fig3E blot/ERK/p-erk-2.tif]

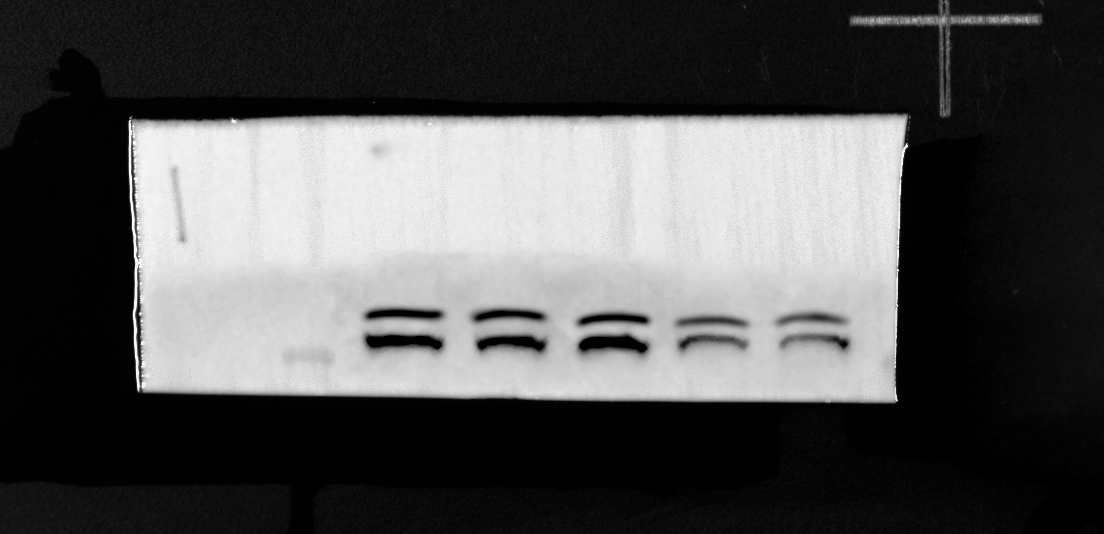

Supplement: Supplemental Information 1 [file peerj-12-17123-s001.zip › Fig3E blot/ERK/p-erk-3.tif]

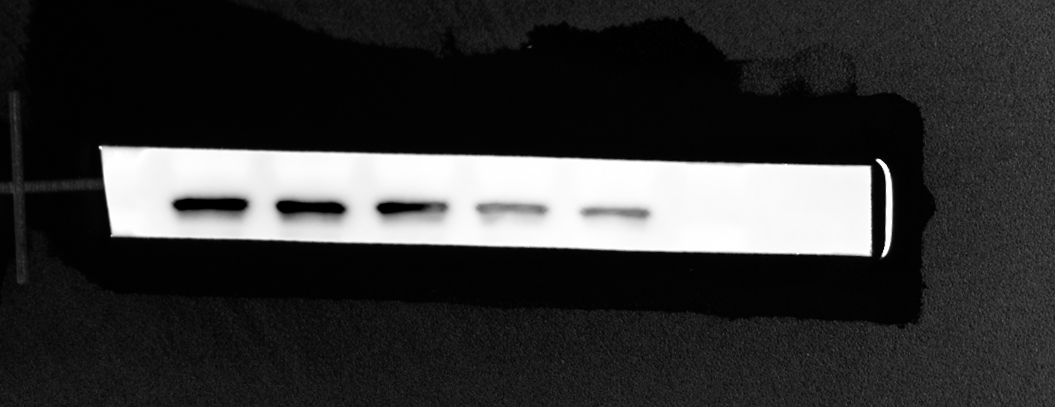

Supplement: Supplemental Information 1 [file peerj-12-17123-s001.zip › Fig3E blot/FGF23/FGF23-1.tif]

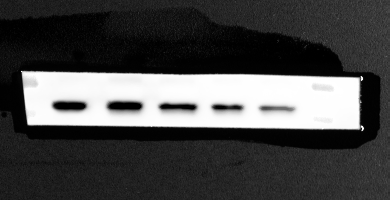

Supplement: Supplemental Information 1 [file peerj-12-17123-s001.zip › Fig3E blot/FGF23/FGF23-2.tif]

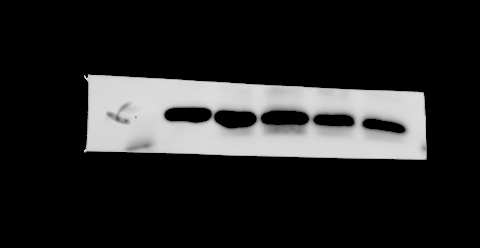

Supplement: Supplemental Information 1 [file peerj-12-17123-s001.zip › Fig3E blot/FGF23/FGF23-3.tif]

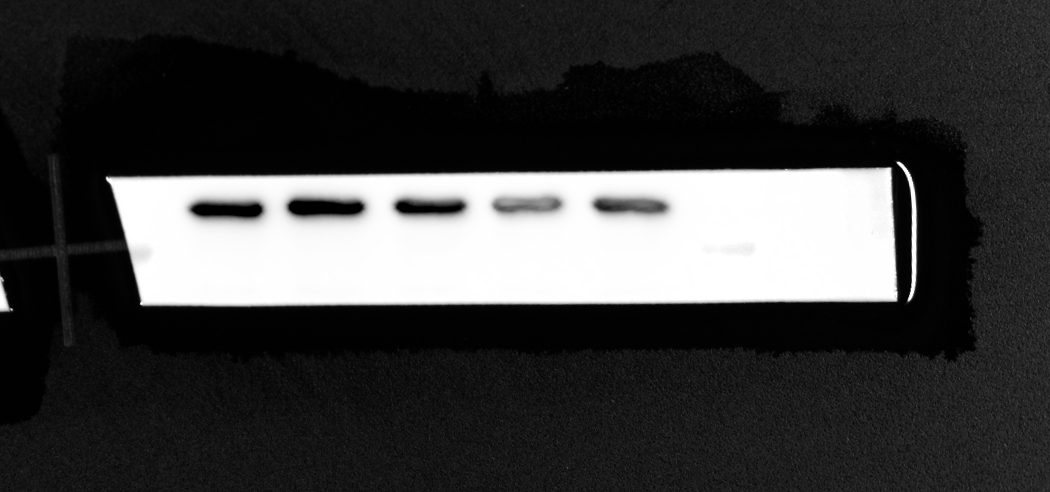

Supplement: Supplemental Information 1 [file peerj-12-17123-s001.zip › Fig3E blot/FGFR4/FGFR4-1.tif]

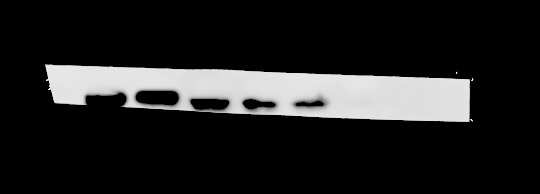

Supplement: Supplemental Information 1 [file peerj-12-17123-s001.zip › Fig3E blot/FGFR4/FGFR4-2.tif]

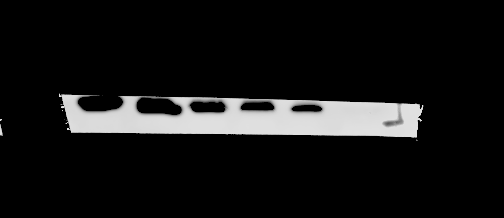

Supplement: Supplemental Information 1 [file peerj-12-17123-s001.zip › Fig3E blot/FGFR4/FGFR4-3.tif]

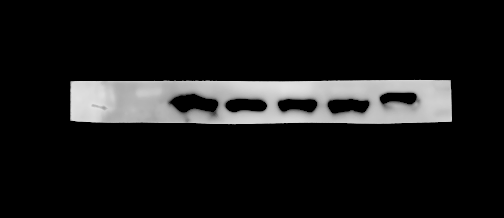

Supplement: Supplemental Information 1 [file peerj-12-17123-s001.zip › Fig3E blot/GAPDH/GAPDH-2.tif]

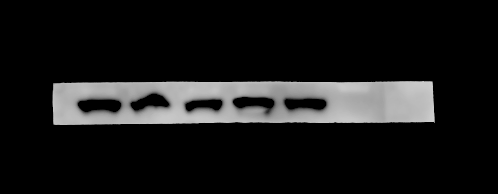

Supplement: Supplemental Information 1 [file peerj-12-17123-s001.zip › Fig3E blot/GAPDH/GAPDH-3.tif]

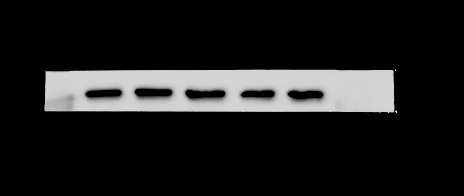

Supplement: Supplemental Information 1 [file peerj-12-17123-s001.zip › Fig3E blot/GAPDH/GAPDH.tif]

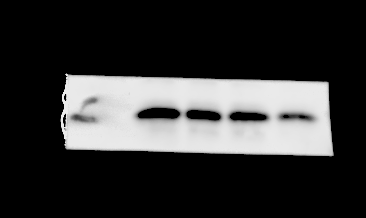

Supplement: Supplemental Information 1 [file peerj-12-17123-s001.zip › Fig3F coip/FGF23-1.tif]

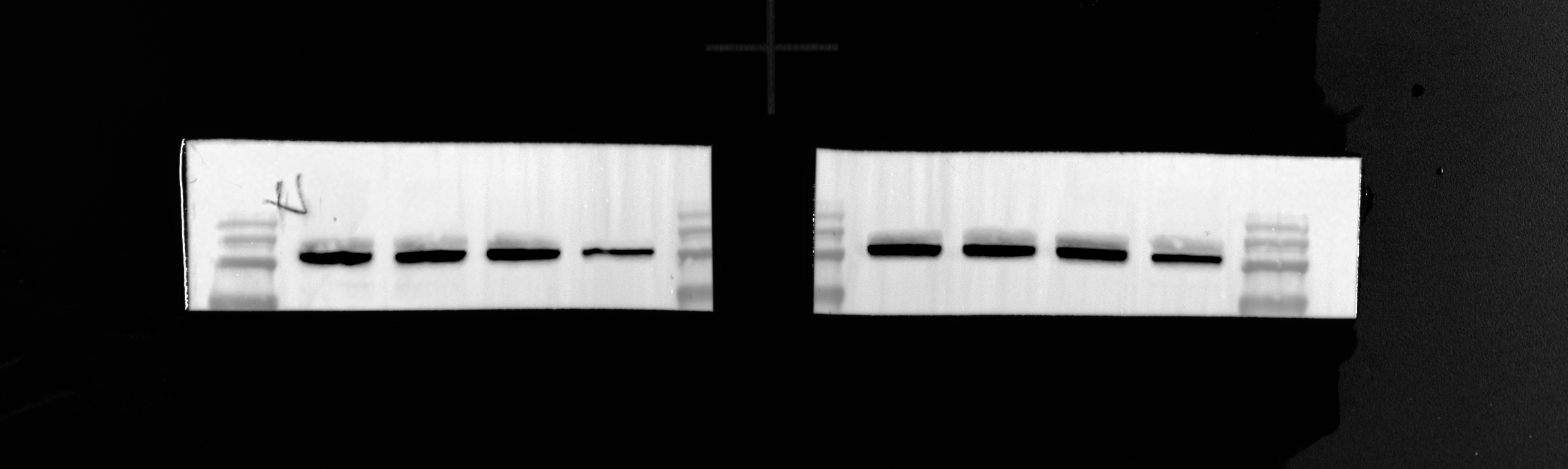

Supplement: Supplemental Information 1 [file peerj-12-17123-s001.zip › Fig3F coip/FGF23-2、3.tif]

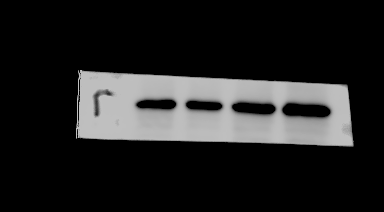

Supplement: Supplemental Information 1 [file peerj-12-17123-s001.zip › Fig3F coip/FGFR4-1.tif]

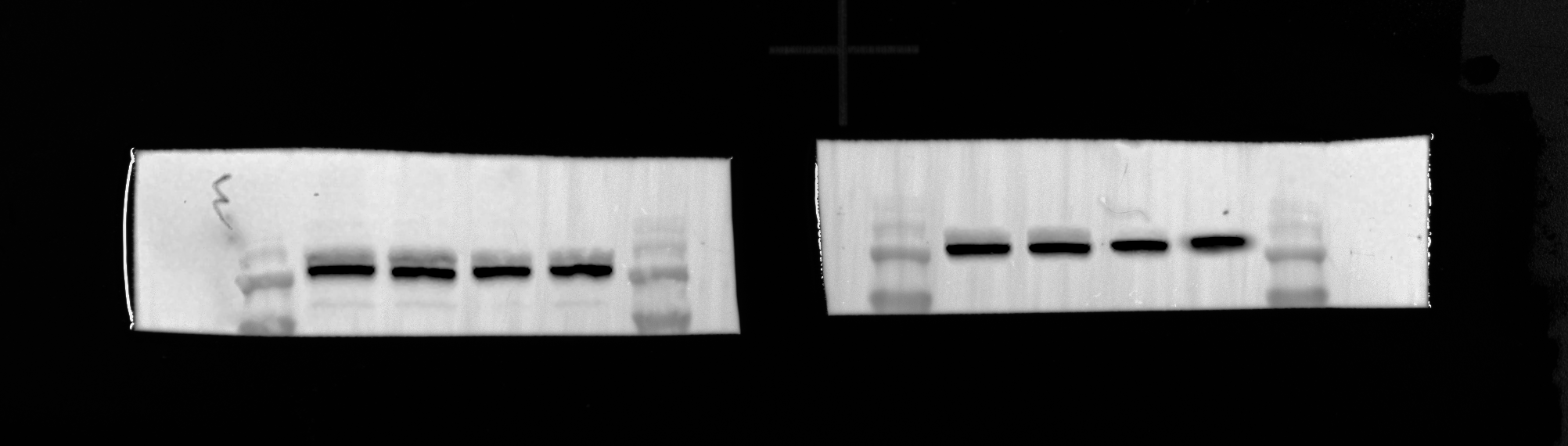

Supplement: Supplemental Information 1 [file peerj-12-17123-s001.zip › Fig3F coip/FGFR4-2、3.tif]

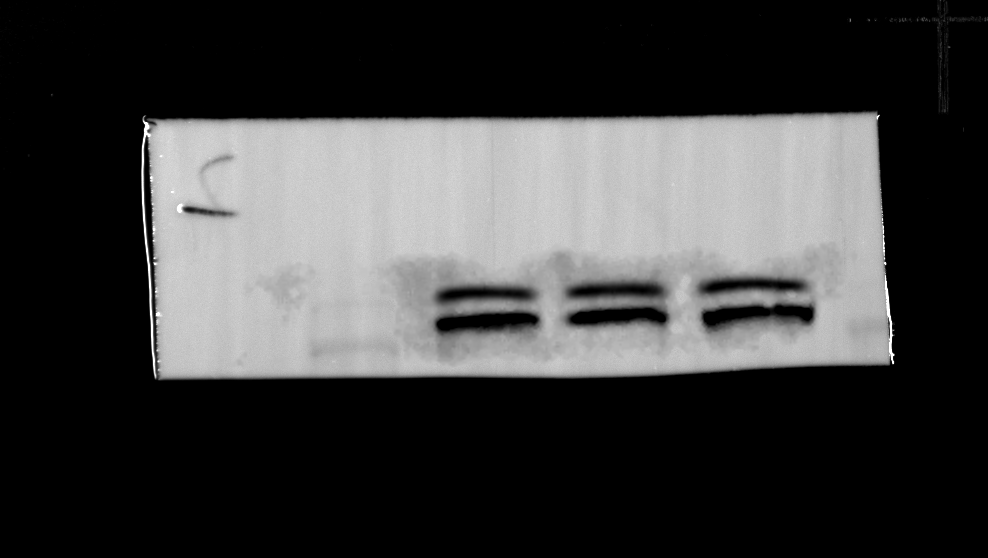

Supplement: Supplemental Information 2 [file peerj-12-17123-s002.zip › Fig4E blot/ERK/ERK-1.tif]

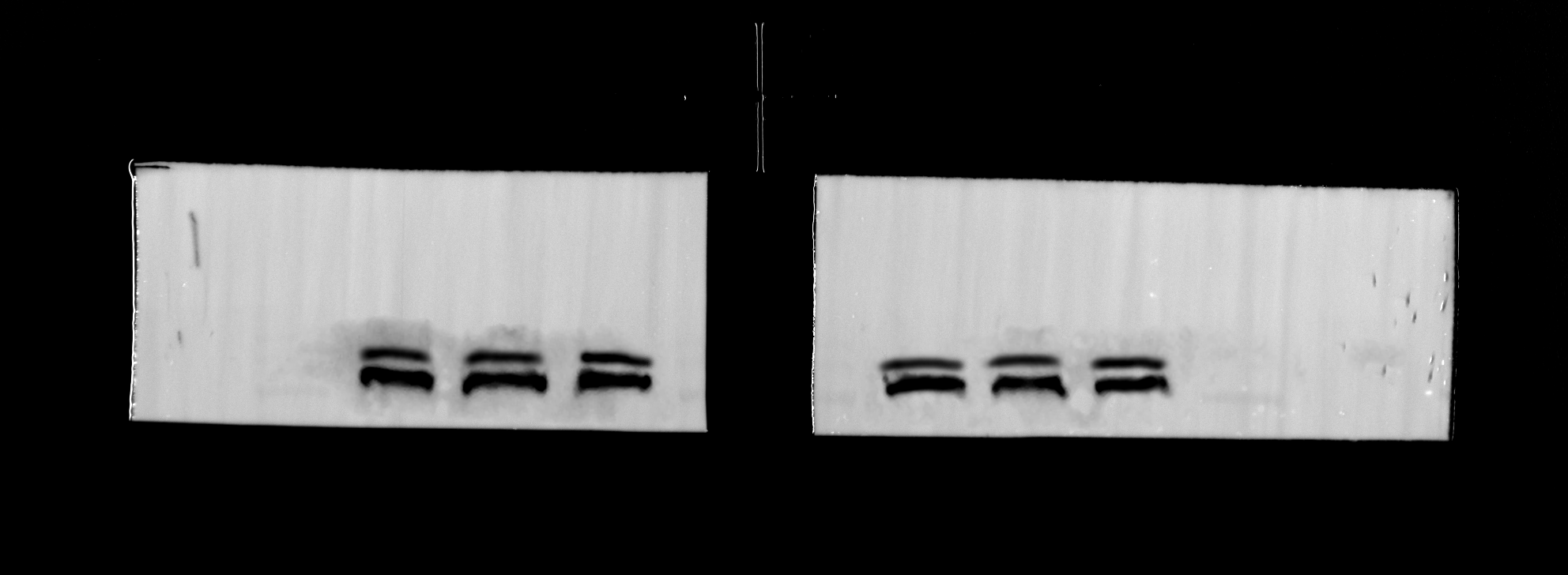

Supplement: Supplemental Information 2 [file peerj-12-17123-s002.zip › Fig4E blot/ERK/ERK-2、3.tif]

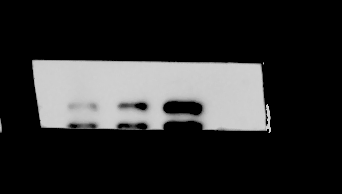

Supplement: Supplemental Information 2 [file peerj-12-17123-s002.zip › Fig4E blot/ERK/p-ERK-1.tif]

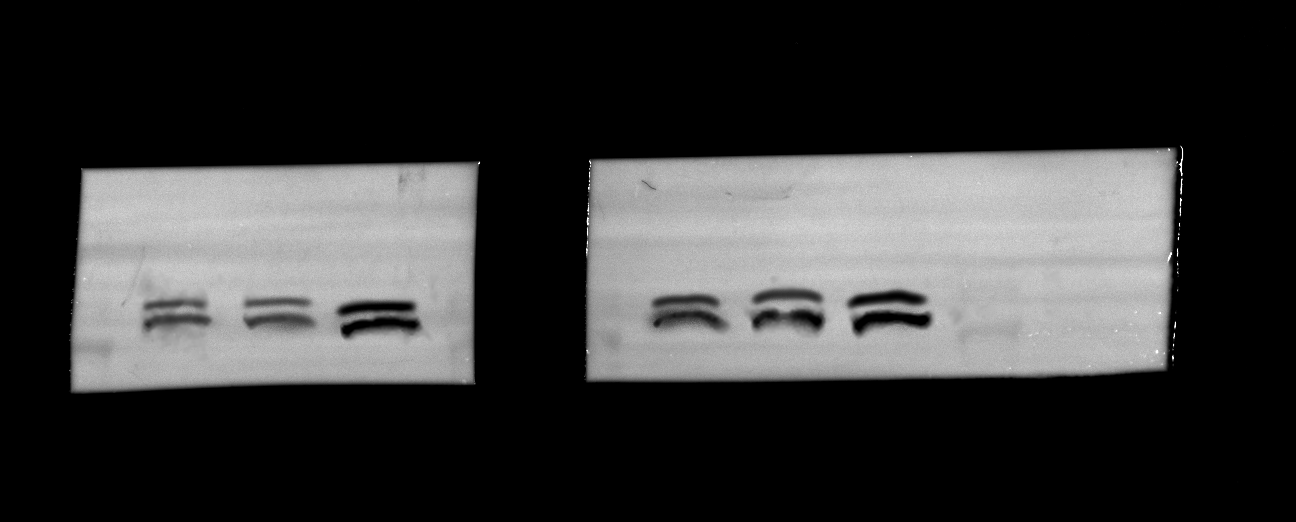

Supplement: Supplemental Information 2 [file peerj-12-17123-s002.zip › Fig4E blot/ERK/p-ERK-2、3.tif]

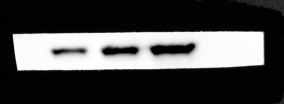

Supplement: Supplemental Information 2 [file peerj-12-17123-s002.zip › Fig4E blot/FGF23/FGF23-1.tif]

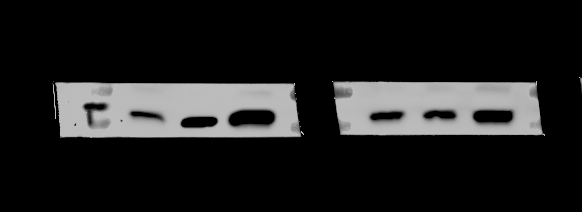

Supplement: Supplemental Information 2 [file peerj-12-17123-s002.zip › Fig4E blot/FGF23/FGF23-2、3.tif]

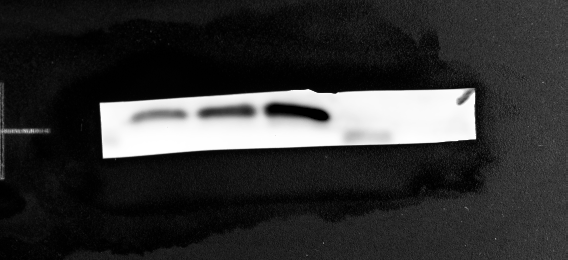

Supplement: Supplemental Information 2 [file peerj-12-17123-s002.zip › Fig4E blot/FGFR4/FGFR4-1.tif]

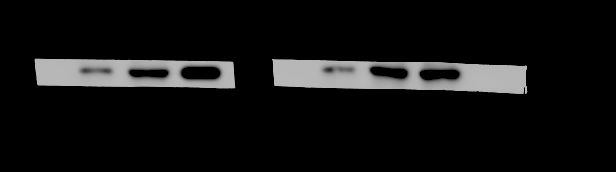

Supplement: Supplemental Information 2 [file peerj-12-17123-s002.zip › Fig4E blot/FGFR4/FGFR4-2、3.tif]

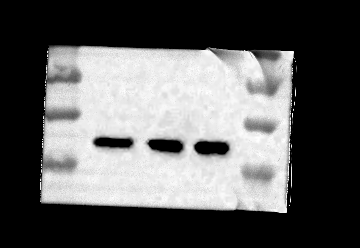

Supplement: Supplemental Information 2 [file peerj-12-17123-s002.zip › Fig4E blot/GAPDH/GAPDH-1.tif]

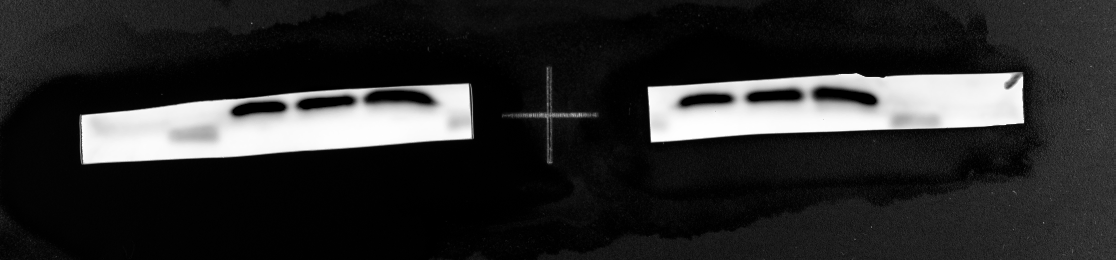

Supplement: Supplemental Information 2 [file peerj-12-17123-s002.zip › Fig4E blot/GAPDH/GAPDH-2、3.tif]

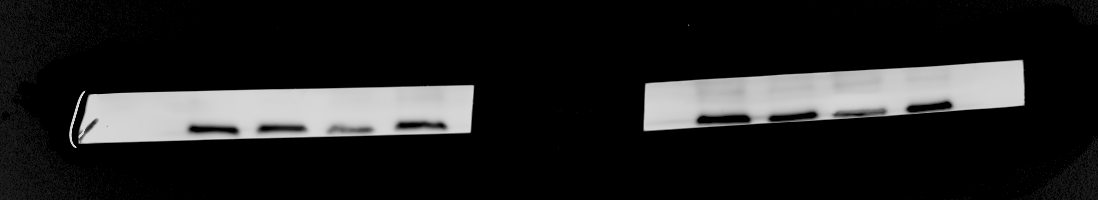

Supplement: Supplemental Information 2 [file peerj-12-17123-s002.zip › Fig4F coip/FGF23-1、2.tif]

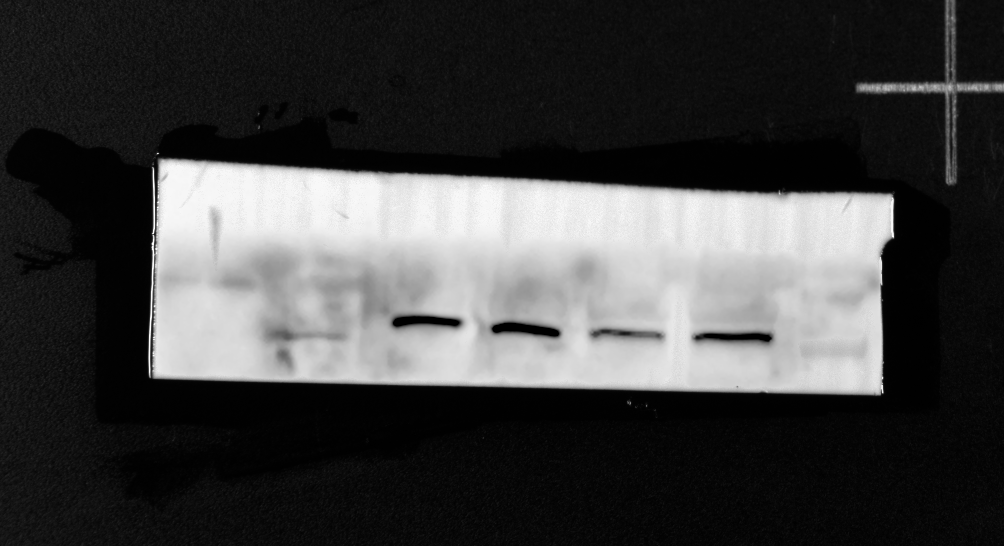

Supplement: Supplemental Information 2 [file peerj-12-17123-s002.zip › Fig4F coip/FGF23-3.tif]

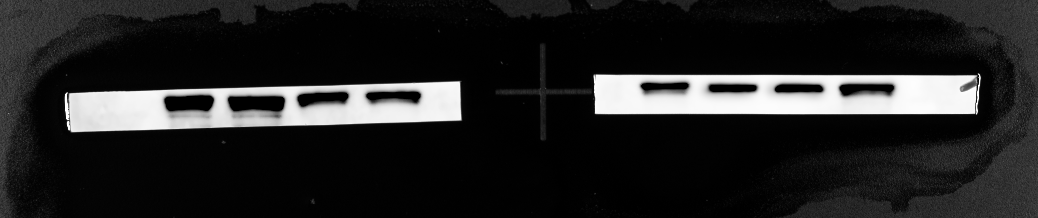

Supplement: Supplemental Information 2 [file peerj-12-17123-s002.zip › Fig4F coip/FGFR4-1、2.tif]

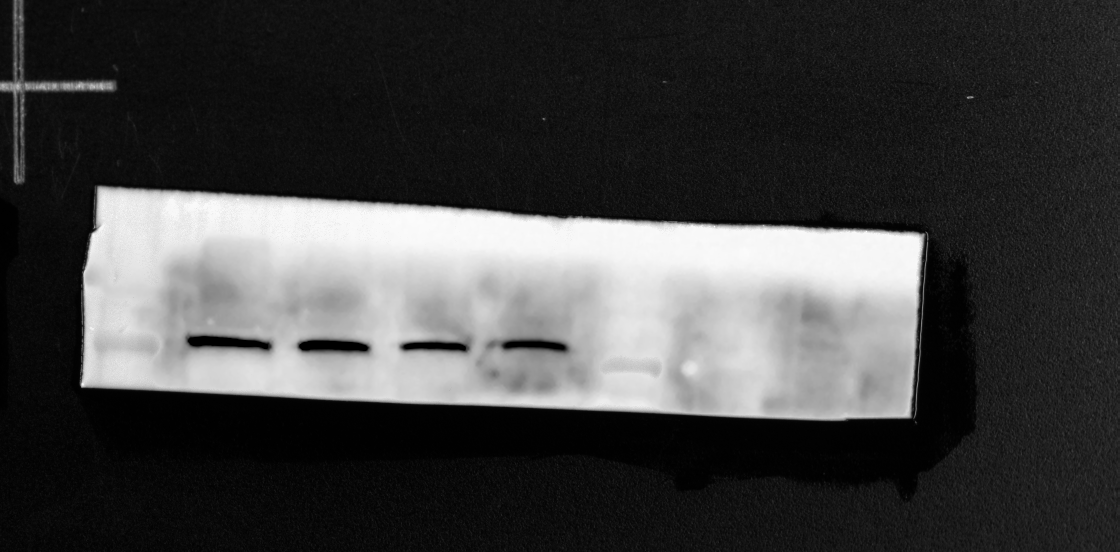

Supplement: Supplemental Information 2 [file peerj-12-17123-s002.zip › Fig4F coip/FGFR4-3.tif]

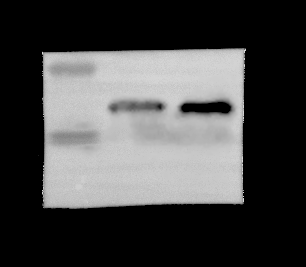

Supplement: Supplemental Information 2 [file peerj-12-17123-s002.zip › Fig7D&E Blot/BAX/BAX-1.tif]

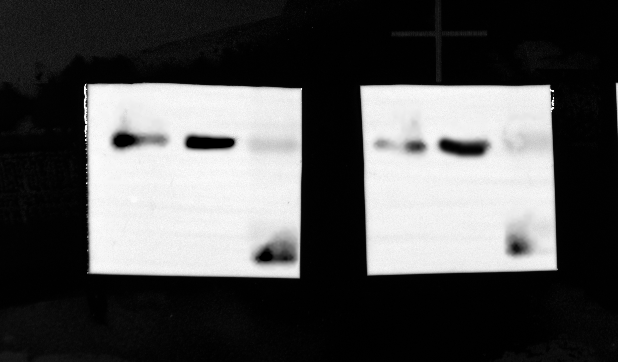

Supplement: Supplemental Information 2 [file peerj-12-17123-s002.zip › Fig7D&E Blot/BAX/BAX-2、3.tif]

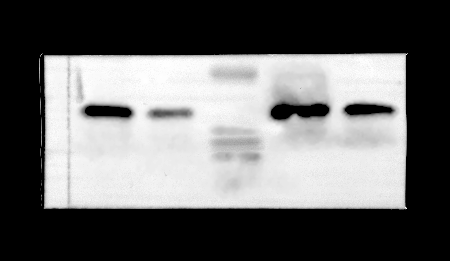

Supplement: Supplemental Information 2 [file peerj-12-17123-s002.zip › Fig7D&E Blot/BCL-2/BCL-2-1、2.tif]

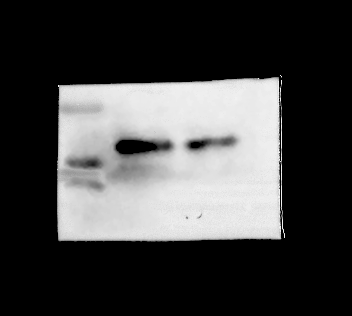

Supplement: Supplemental Information 2 [file peerj-12-17123-s002.zip › Fig7D&E Blot/BCL-2/BCL-2-3.tif]

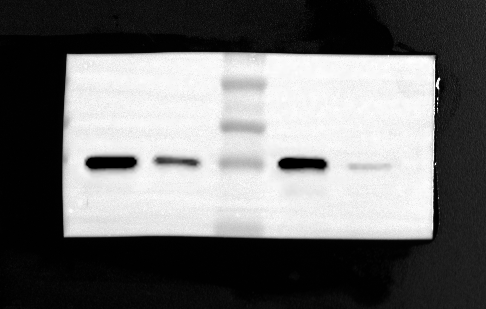

Supplement: Supplemental Information 2 [file peerj-12-17123-s002.zip › Fig7D&E Blot/EPO/EPO-1、2.tif]

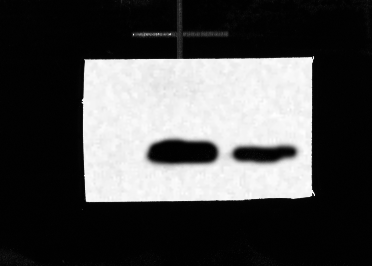

Supplement: Supplemental Information 2 [file peerj-12-17123-s002.zip › Fig7D&E Blot/EPO/EPO-3.tif]

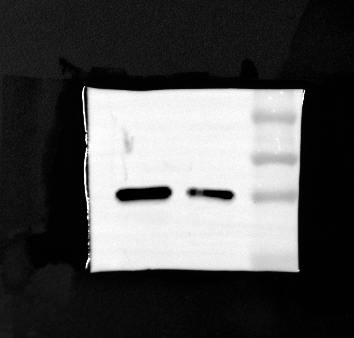

Supplement: Supplemental Information 2 [file peerj-12-17123-s002.zip › Fig7D&E Blot/FGF23/FGF23-1.tif]

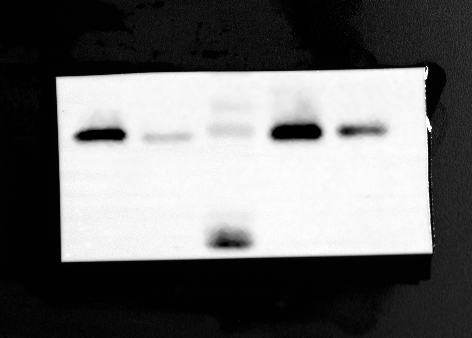

Supplement: Supplemental Information 2 [file peerj-12-17123-s002.zip › Fig7D&E Blot/FGF23/FGF23-2、3.tif]

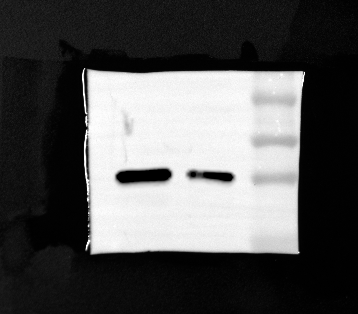

Supplement: Supplemental Information 2 [file peerj-12-17123-s002.zip › Fig7D&E Blot/FGFR4/FGFR4-1.tif]

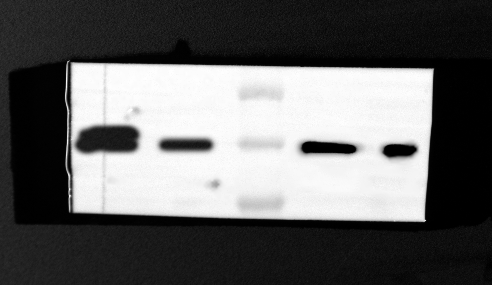

Supplement: Supplemental Information 2 [file peerj-12-17123-s002.zip › Fig7D&E Blot/FGFR4/FGFR4-2、3.tif]

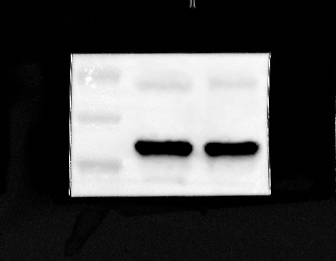

Supplement: Supplemental Information 2 [file peerj-12-17123-s002.zip › Fig7D&E Blot/GAPDH1/GAPDH1-1.tif]

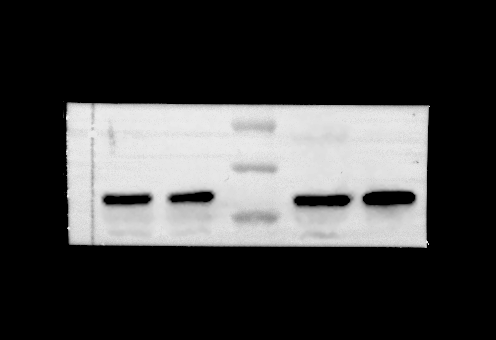

Supplement: Supplemental Information 2 [file peerj-12-17123-s002.zip › Fig7D&E Blot/GAPDH1/GAPDH1-2、3.tif]

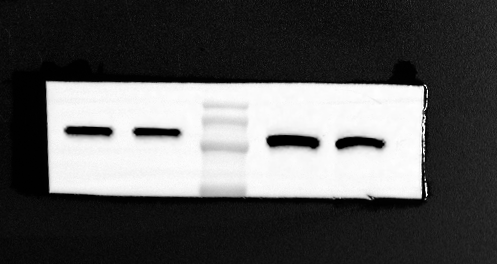

Supplement: Supplemental Information 2 [file peerj-12-17123-s002.zip › Fig7D&E Blot/GAPDH2/GAPDH-1、2.tif]

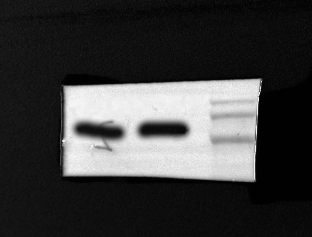

Supplement: Supplemental Information 2 [file peerj-12-17123-s002.zip › Fig7D&E Blot/GAPDH2/GAPDH-3.tif]

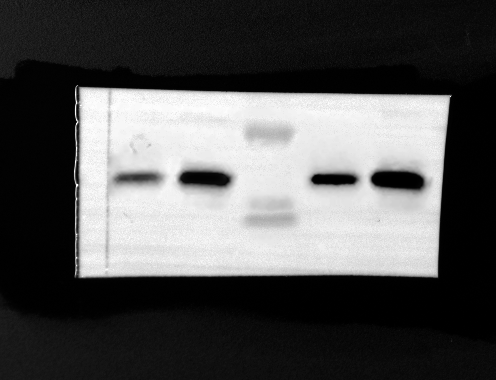

Supplement: Supplemental Information 2 [file peerj-12-17123-s002.zip › Fig7D&E Blot/caspase-3/caspase-3-1、2.tif]

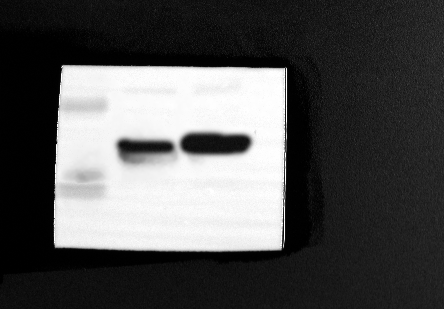

Supplement: Supplemental Information 2 [file peerj-12-17123-s002.zip › Fig7D&E Blot/caspase-3/caspase-3-3.tif]

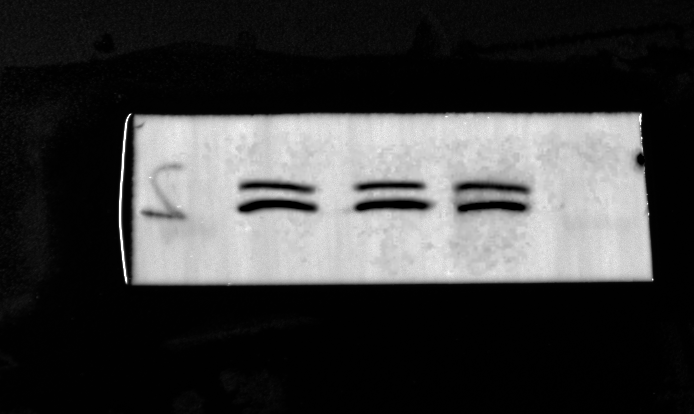

Supplement: Supplemental Information 2 [file peerj-12-17123-s002.zip › FigS1 OE blot/ERK/erk-3.tif]

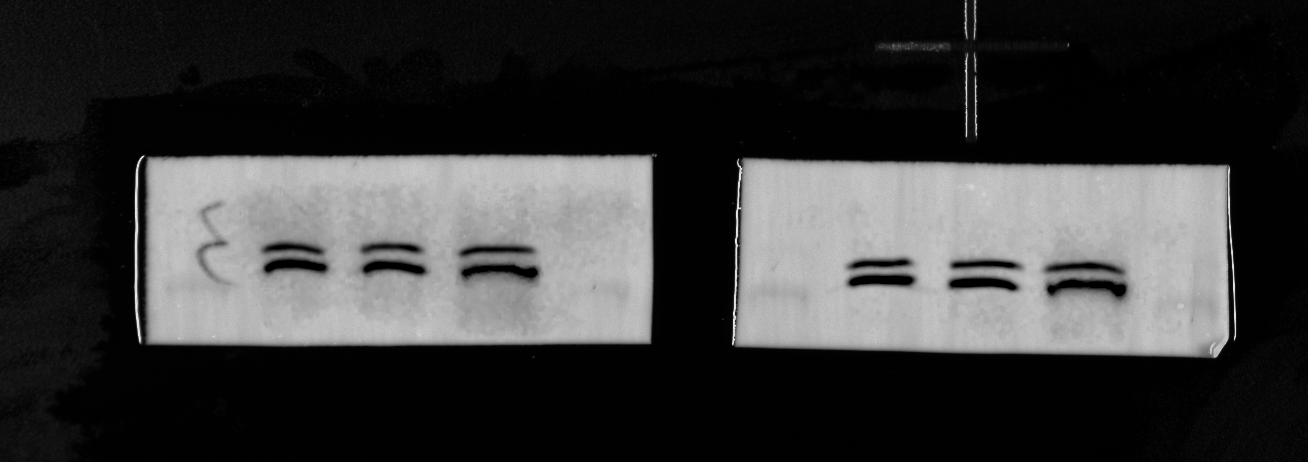

Supplement: Supplemental Information 2 [file peerj-12-17123-s002.zip › FigS1 OE blot/ERK/erk1、2.tif]

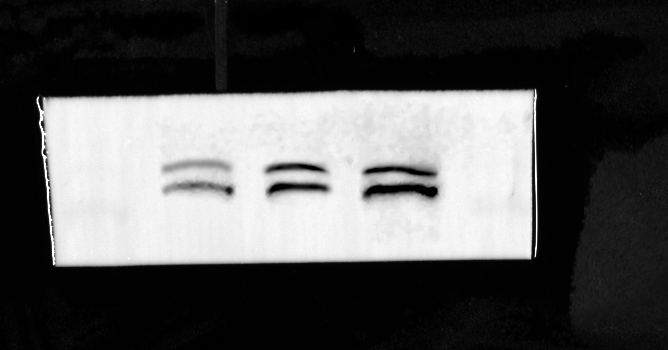

Supplement: Supplemental Information 2 [file peerj-12-17123-s002.zip › FigS1 OE blot/ERK/p-erk-3.tif]

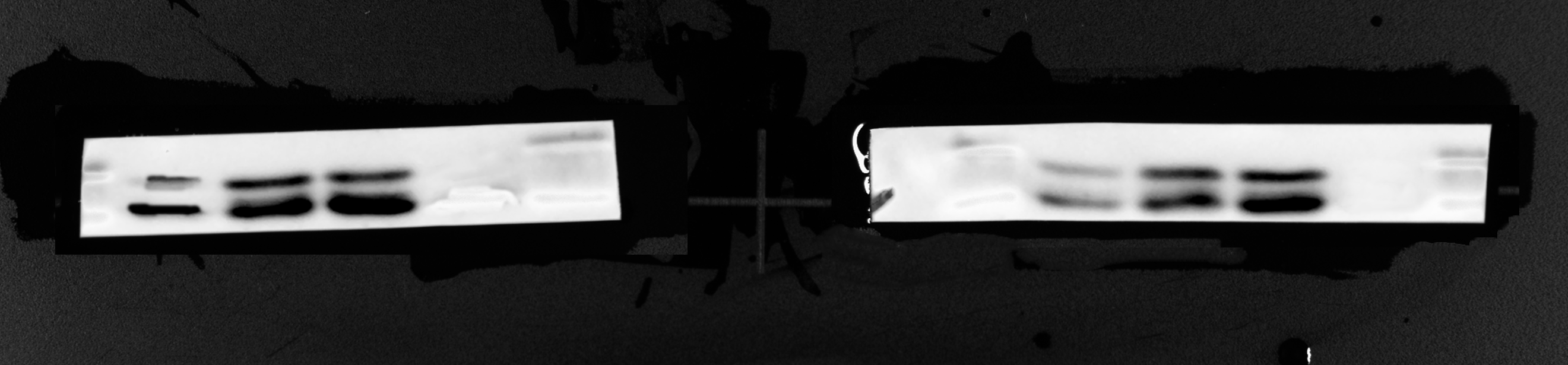

Supplement: Supplemental Information 2 [file peerj-12-17123-s002.zip › FigS1 OE blot/ERK/p-erk1、2.tif]

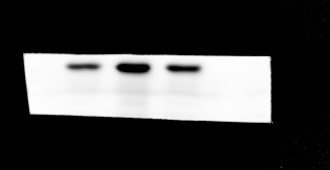

Supplement: Supplemental Information 2 [file peerj-12-17123-s002.zip › FigS1 OE blot/FGF23/FGF23-1.tif]

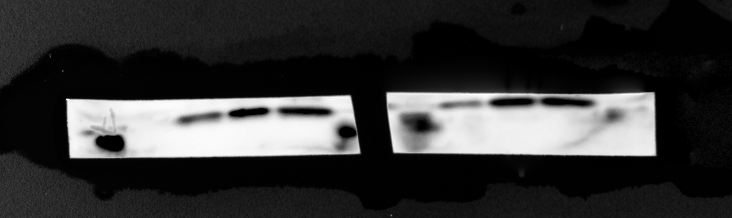

Supplement: Supplemental Information 2 [file peerj-12-17123-s002.zip › FigS1 OE blot/FGF23/FGF23-2、3.tif]

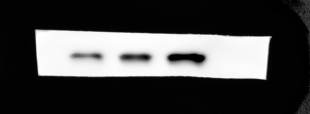

Supplement: Supplemental Information 2 [file peerj-12-17123-s002.zip › FigS1 OE blot/FGFR4/FGFR4-1.tif]

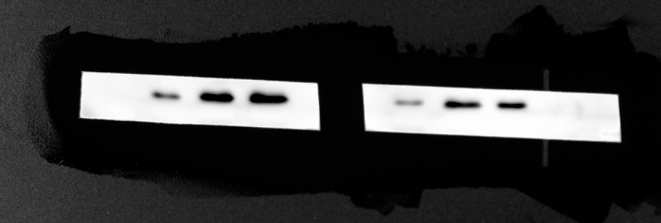

Supplement: Supplemental Information 2 [file peerj-12-17123-s002.zip › FigS1 OE blot/FGFR4/FGFR4-2、3.tif]

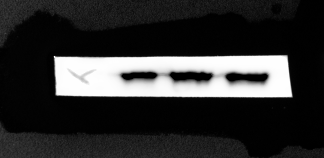

Supplement: Supplemental Information 2 [file peerj-12-17123-s002.zip › FigS1 OE blot/GAPDH/GAPDH-1.tif]

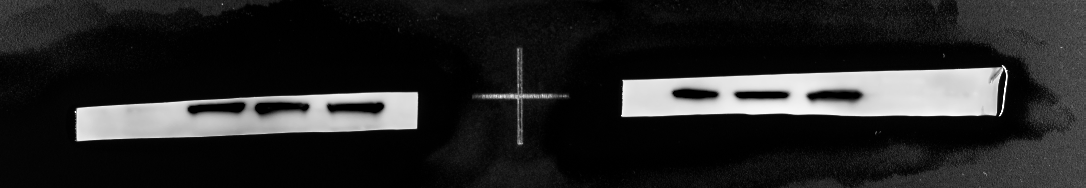

Supplement: Supplemental Information 2 [file peerj-12-17123-s002.zip › FigS1 OE blot/GAPDH/GAPDH-2、3.tif]

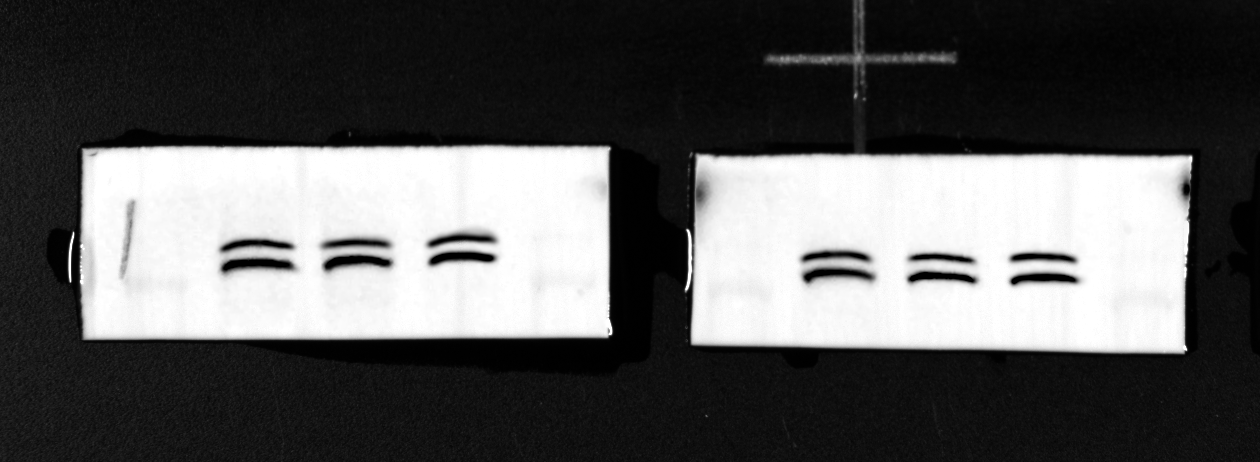

Supplement: Supplemental Information 2 [file peerj-12-17123-s002.zip › FigS1 si blot/ERK/erk-1、2.tif]

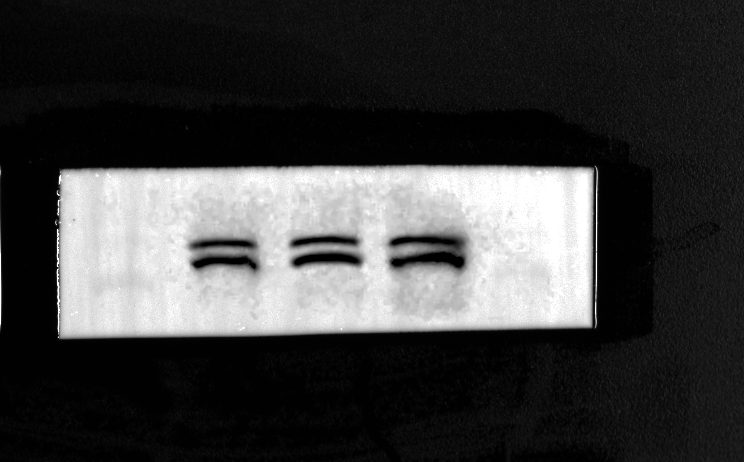

Supplement: Supplemental Information 2 [file peerj-12-17123-s002.zip › FigS1 si blot/ERK/erk-3.tif]

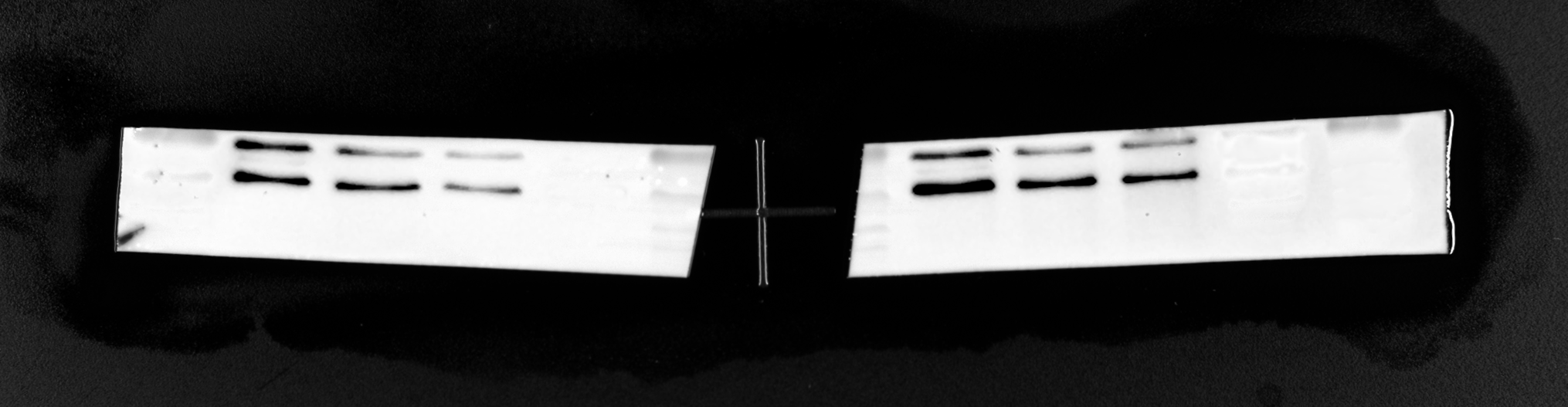

Supplement: Supplemental Information 2 [file peerj-12-17123-s002.zip › FigS1 si blot/ERK/p-erk-1、2.tif]

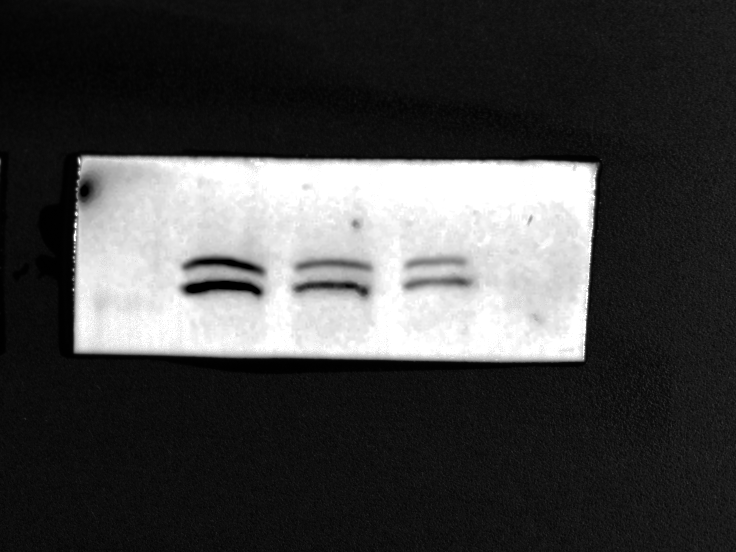

Supplement: Supplemental Information 2 [file peerj-12-17123-s002.zip › FigS1 si blot/ERK/p-erk-3.tif]

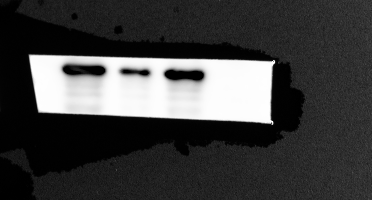

Supplement: Supplemental Information 2 [file peerj-12-17123-s002.zip › FigS1 si blot/FGF23/FGF23-1.tif]

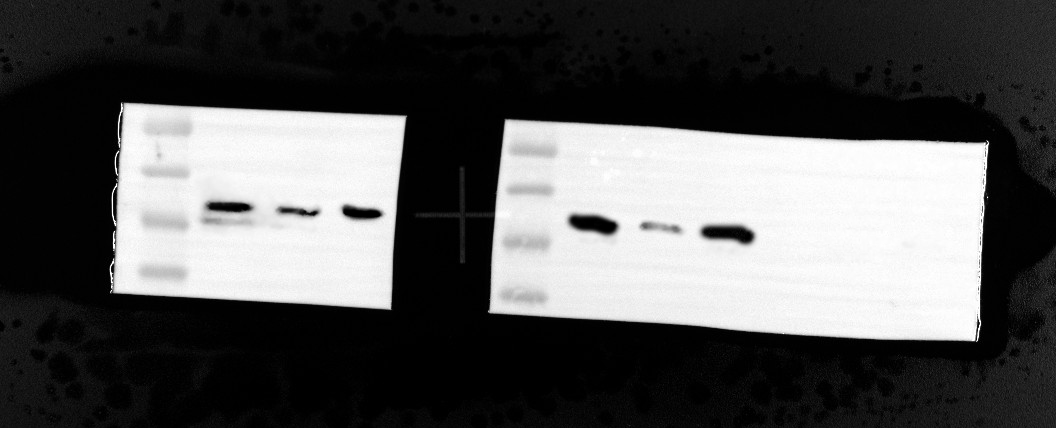

Supplement: Supplemental Information 2 [file peerj-12-17123-s002.zip › FigS1 si blot/FGF23/FGF23-2、3.tif]

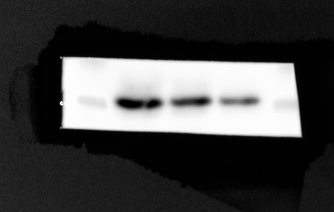

Supplement: Supplemental Information 2 [file peerj-12-17123-s002.zip › FigS1 si blot/FGFR4/FGFR4-1.tif]

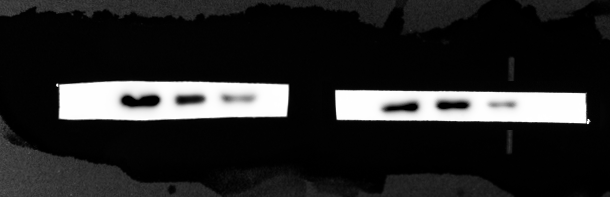

Supplement: Supplemental Information 2 [file peerj-12-17123-s002.zip › FigS1 si blot/FGFR4/FGFR4-2、3.tif]

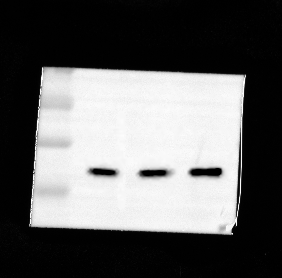

Supplement: Supplemental Information 2 [file peerj-12-17123-s002.zip › FigS1 si blot/GAPDH/GAPDH-1.tif]

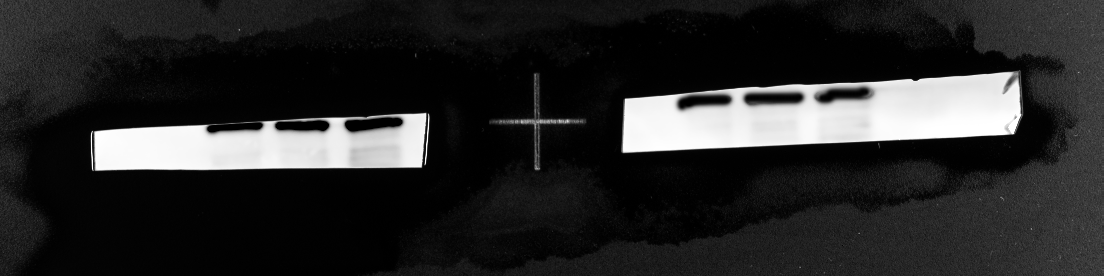

Supplement: Supplemental Information 2 [file peerj-12-17123-s002.zip › FigS1 si blot/GAPDH/GAPDH-2、3.tif]

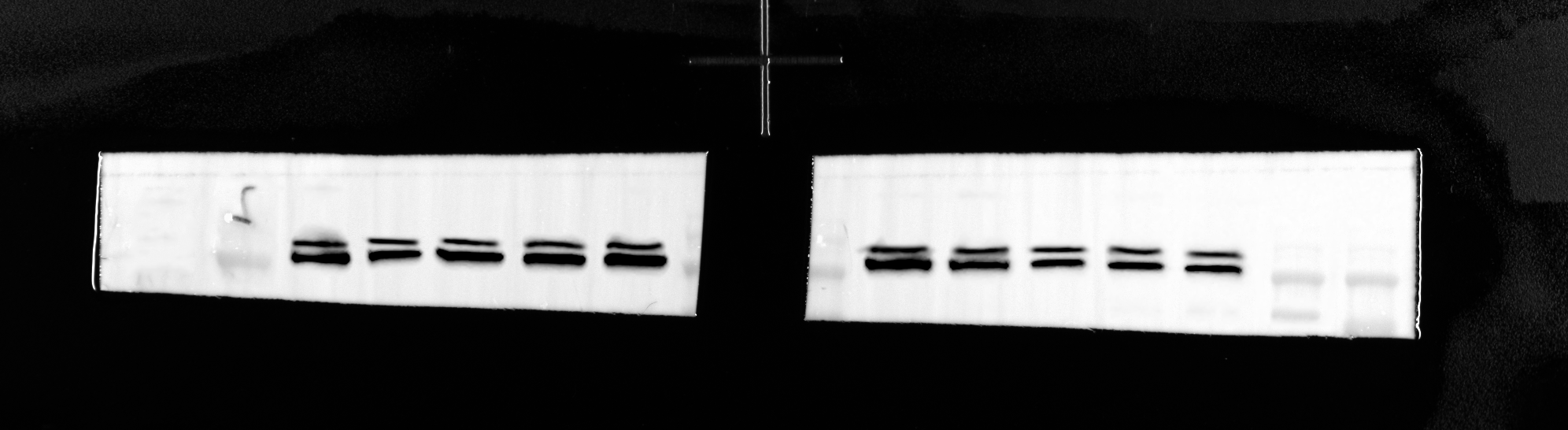

Supplement: Supplemental Information 2 [file peerj-12-17123-s002.zip › FigS2B blot/ERK/erk-1、2.tif]

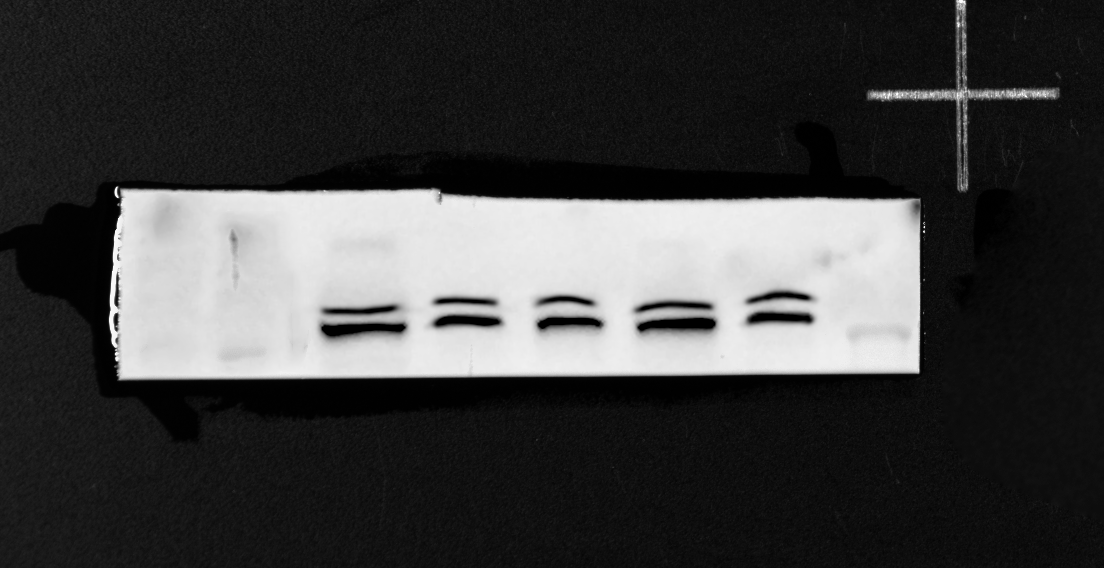

Supplement: Supplemental Information 2 [file peerj-12-17123-s002.zip › FigS2B blot/ERK/erk-3.tif]

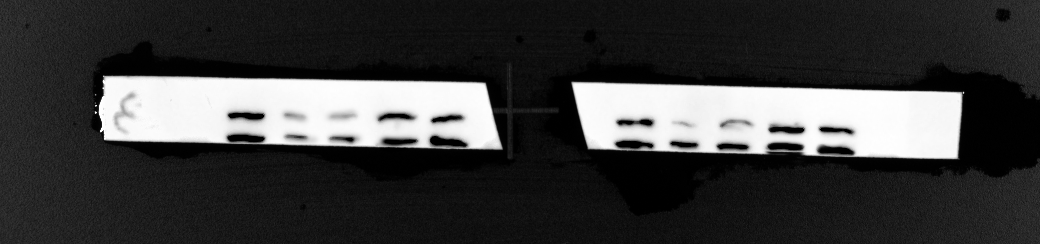

Supplement: Supplemental Information 2 [file peerj-12-17123-s002.zip › FigS2B blot/ERK/p-erk-1、2.tif]

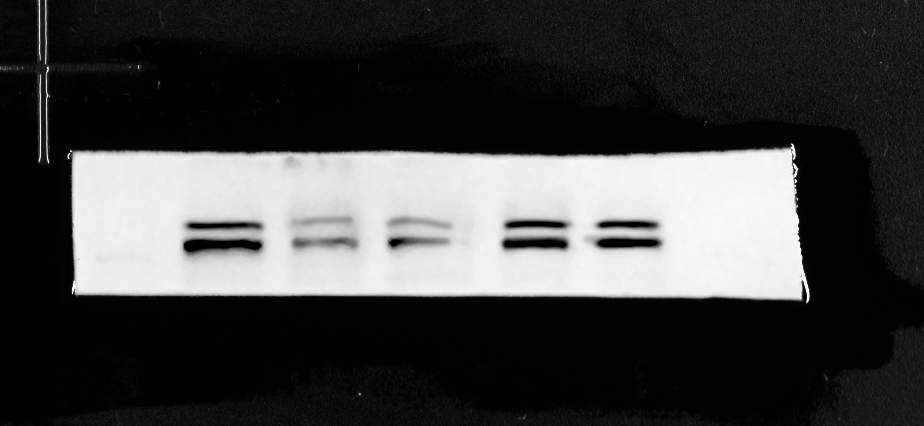

Supplement: Supplemental Information 2 [file peerj-12-17123-s002.zip › FigS2B blot/ERK/p-erk-3.tif]

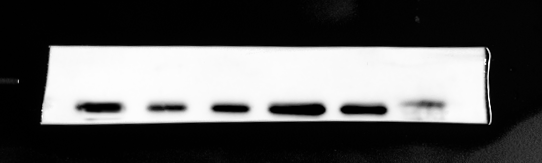

Supplement: Supplemental Information 2 [file peerj-12-17123-s002.zip › FigS2B blot/FGF23/FGF23-1.tif]

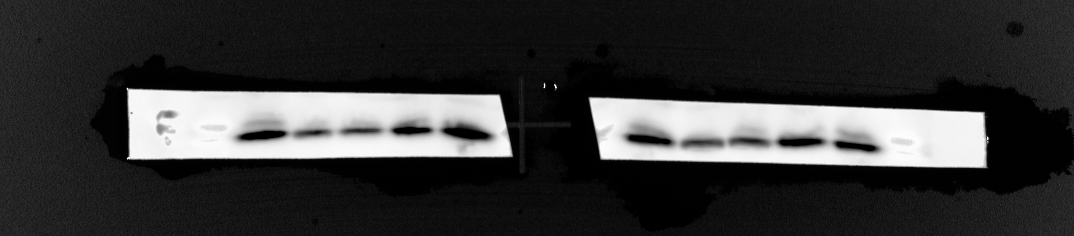

Supplement: Supplemental Information 2 [file peerj-12-17123-s002.zip › FigS2B blot/FGF23/FGF23-2、3.tif]

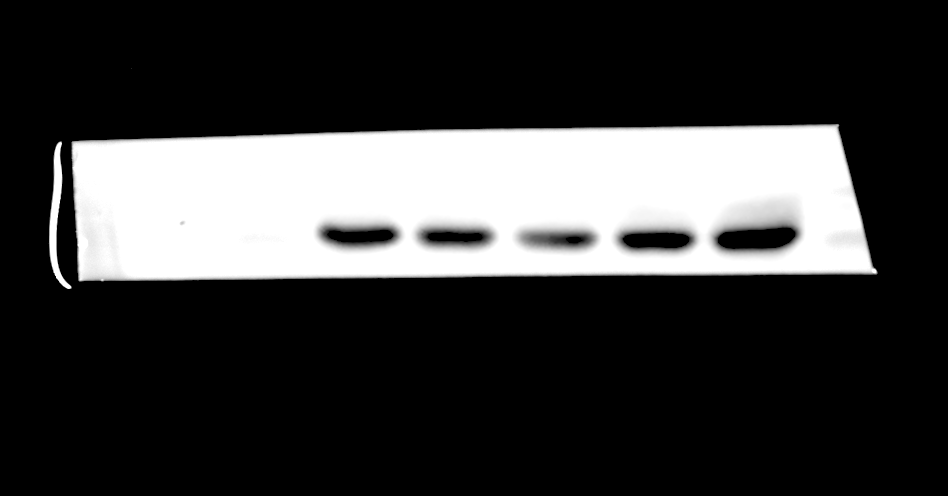

Supplement: Supplemental Information 2 [file peerj-12-17123-s002.zip › FigS2B blot/FGFR4/FGFR4-1.tif]

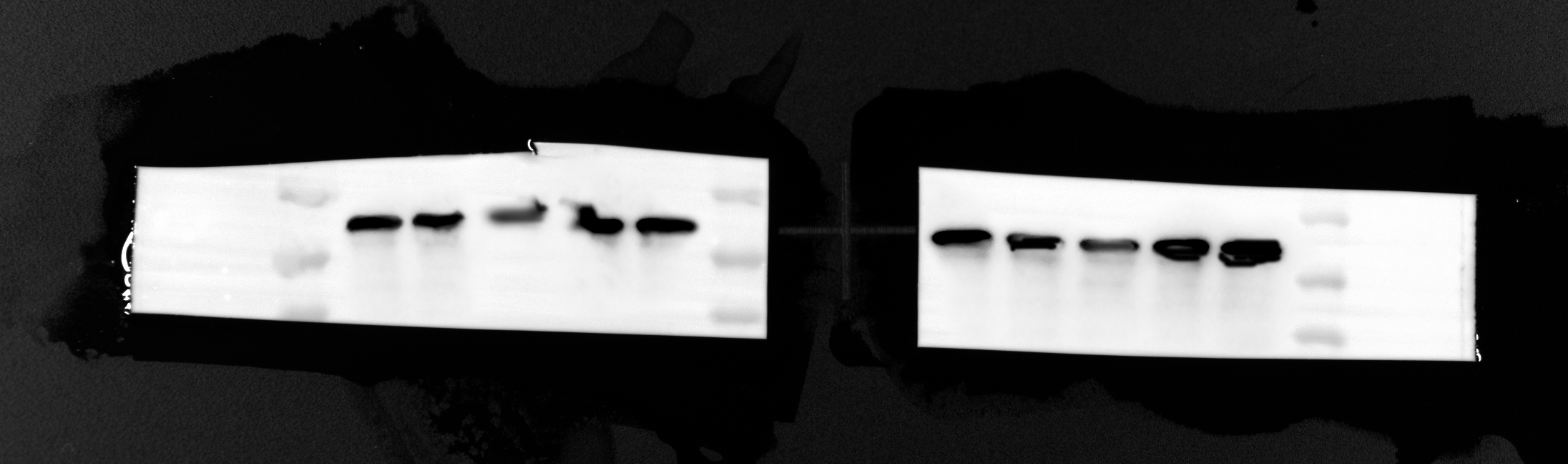

Supplement: Supplemental Information 2 [file peerj-12-17123-s002.zip › FigS2B blot/FGFR4/FGFR4-2、3.tif]

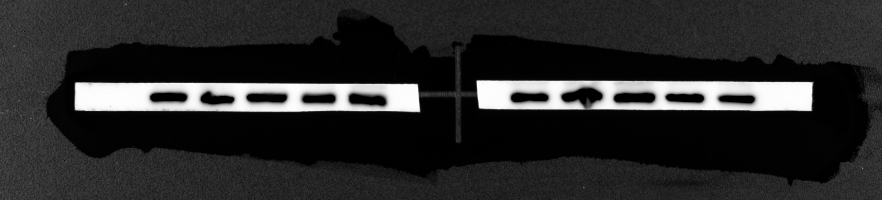

Supplement: Supplemental Information 2 [file peerj-12-17123-s002.zip › FigS2B blot/GAPDH/GAPDH-1、2.tif]
