# Supplementary figures and images for: Erythropoietin alleviates lung ischemia-reperfusion injury by activating the FGF23/FGFR4/ERK signaling pathway (part 2 of 2)
Source: PeerJ. 2024 Mar 27;12:e17123. doi: 10.7717/peerj.17123 (PMC10981413; doi:10.7717/peerj.17123)

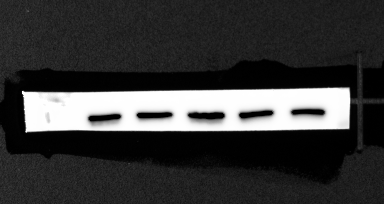

Supplement: Supplemental Information 2 [file peerj-12-17123-s002.zip › FigS2B blot/GAPDH/GAPDH-3.tif]

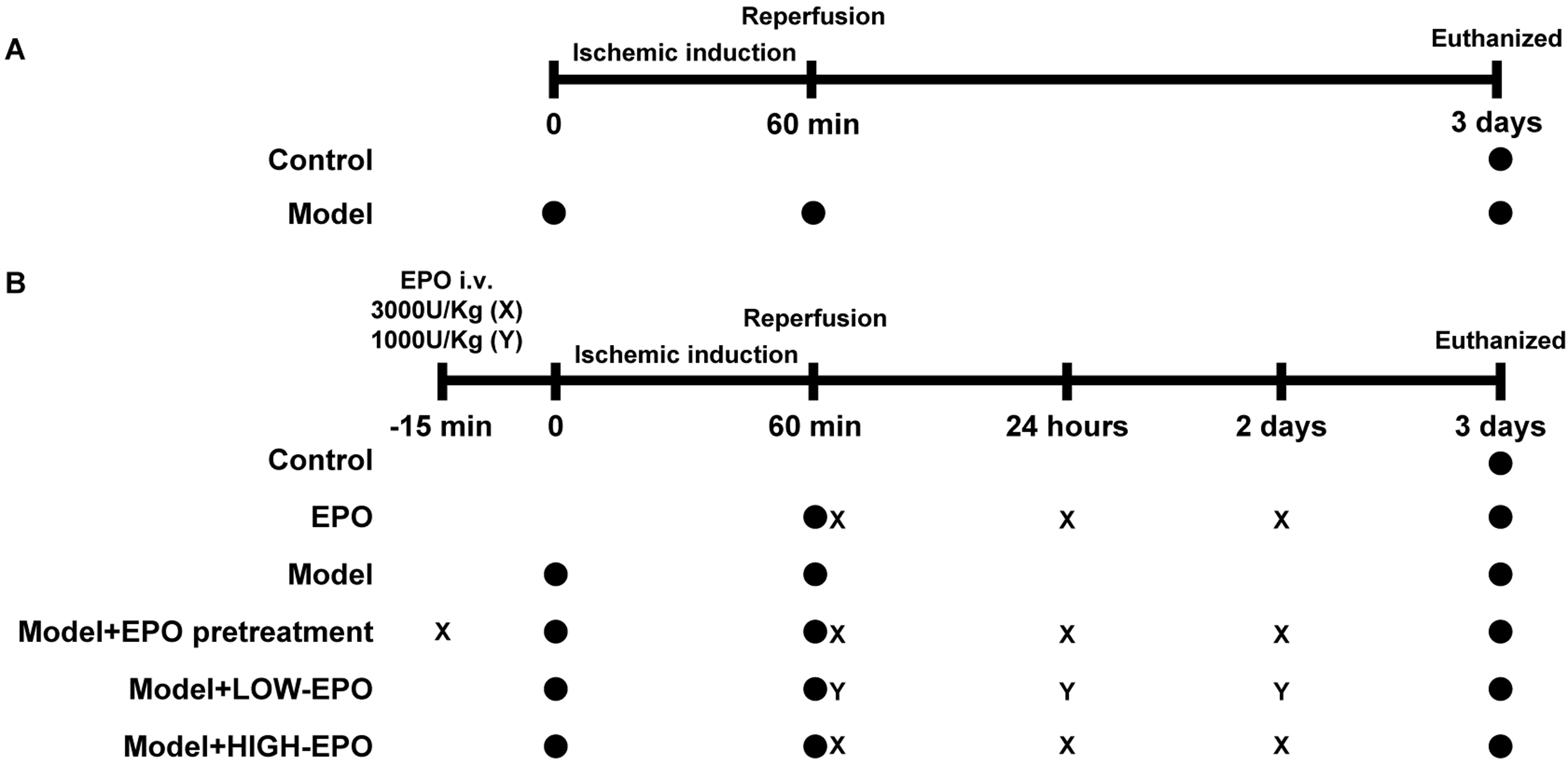

Supplement: Supplemental Information 5 — (A) The control group and the LIRI group (Model group) were established. LIRI was induced through 60 min of ischemia by ligating the left pulmonary hilus, followed by 72 h of reperfusion. (B) The rats grouped as follow: control group, EPO group, Model group, Model + EPO prevention group, Model + LOW-EPO group, Model + HIGH-EPO group. The LIRI was induced though 60 min ischemia by ligation of the left pulmonary hilus and 72 h reperfusion. Solid circles represent the operations corresponding to the timeline above. “X” and “Y” respectively represent the caudal vein injections of EPO at dosages of 3,000 U/kg and 1,000 U/kg. [file peerj-12-17123-s005.png]

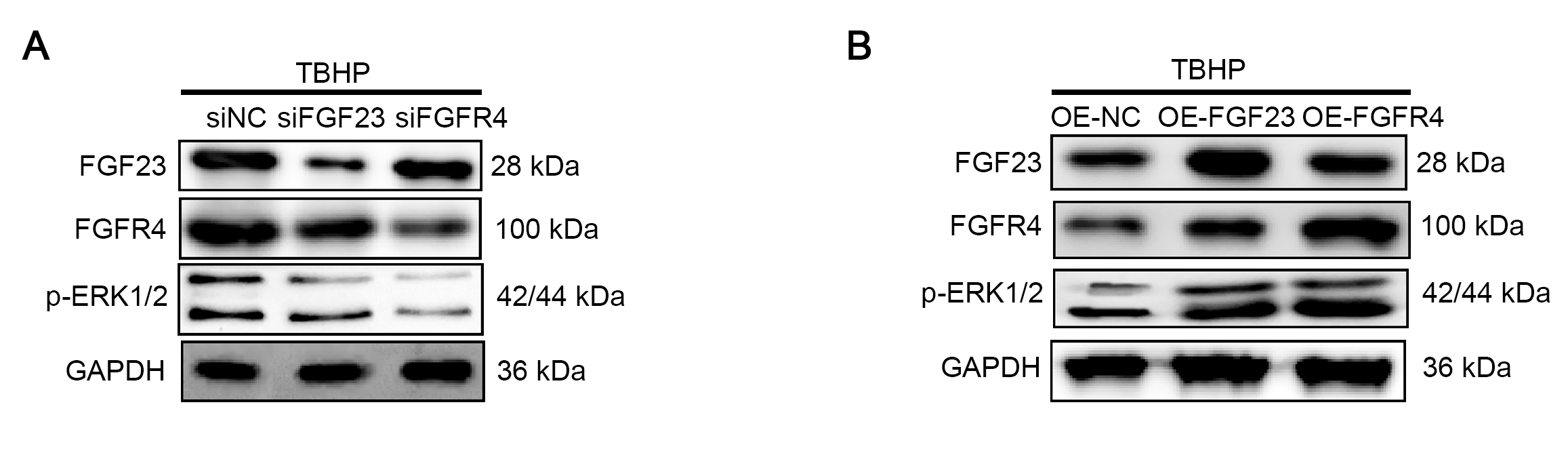

Supplement: Supplemental Information 6 — (A) siFGF, siFGFR4 and siNC were transfected into TBHP-induced BEAS-2B cells. The expression level of FGF23, FGFR4 and p-ERK1/2 was examined by Western blot. (B) OE-FGF, OE-FGFR4 and OE-NC were transfected into TBHP-induced BEAS-2B cells. The expression level of FGF23, FGFR4 and p-ERK1/2 was monitored by Western blot. [file peerj-12-17123-s006.png]

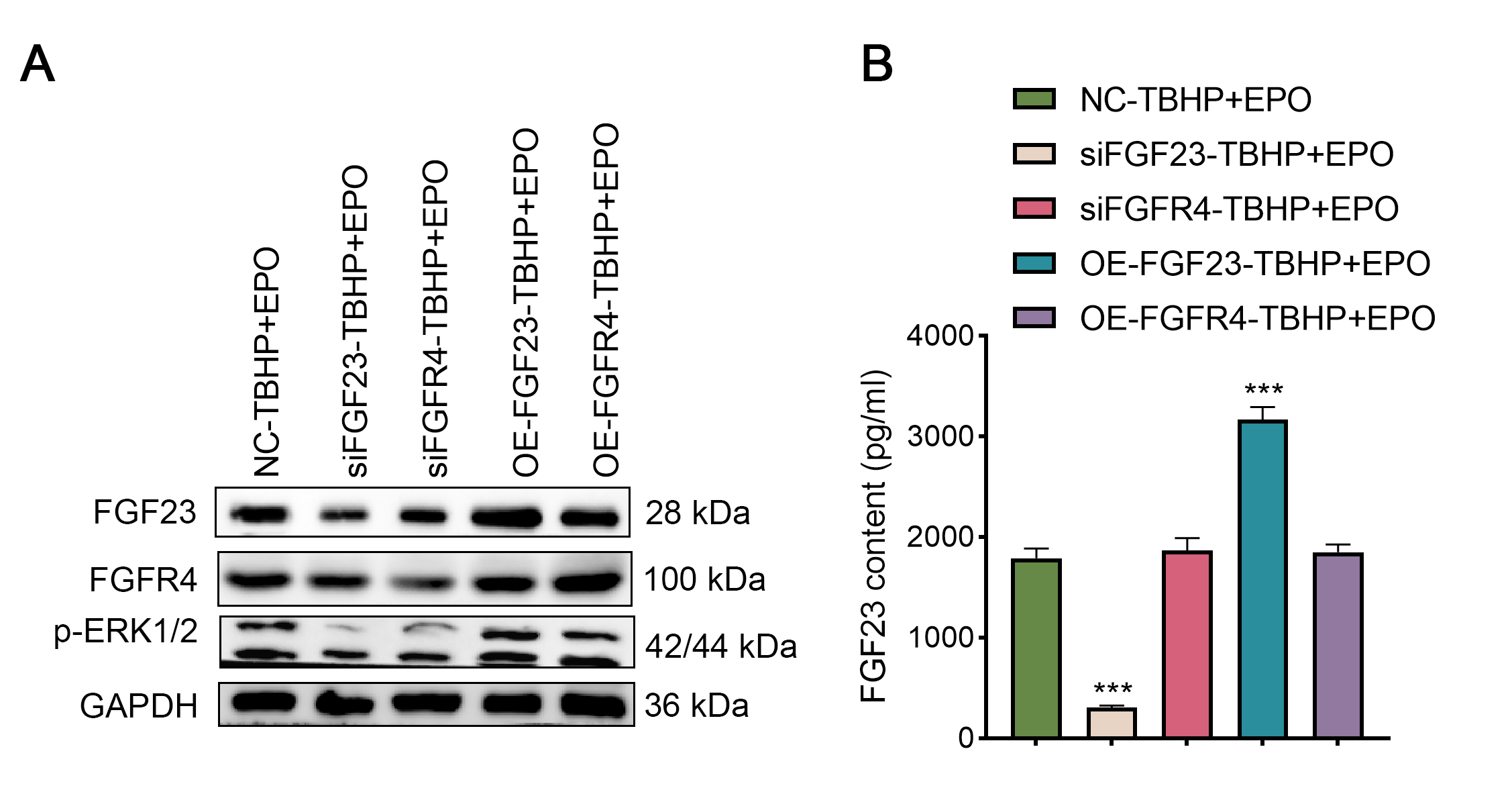

Supplement: Supplemental Information 7 — (A) siFGF, siFGFR4, OE-FGFR4, OE-FGF23 and its control were transfected into TBHP and EPO-induced BEAS-2B cells. The expression level of FGF23, FGFR4 and p-ERK1/2 was examined by Western blot. (B) The expression level of FGF23 in the serum of patients with LIRI was examined by Western blot. [file peerj-12-17123-s007.png]
