# Supplementary material for: Erythropoietin alleviates lung ischemia-reperfusion injury by activating the FGF23/FGFR4/ERK signaling pathway
Source: PeerJ. 2024 Mar 27;12:e17123. doi: 10.7717/peerj.17123 (PMC10981413; doi:10.7717/peerj.17123)
Supplement: Supplemental Information 3 [file peerj-12-17123-s003.zip › English-language codebook.docx]

| 标准品浓度 | Standard concentration |
| --- | --- |
| 样本名称 | Sample Name |
| 复孔 | Repeat hole |
| 平均 | average |
| 样本 | sample |
| 零孔 | zero pore |
| 稀释倍数 | Times of dilution |
| 样品浓度 | Sample concentration |
| 浓度拟合曲线 | Concentration fitting curve |
| 血清 | serum |
| 引物名称 | Primer name |
| 引物序列 | Primer sequence |
| 分析 | analysis |
| 原始数据 | Original data |
